# Improved support vector machine classification algorithm based on adaptive feature weight updating in the Hadoop cluster environment

**DOI:** 10.1371/journal.pone.0215136

**Published:** 2019-04-10

**Authors:** Jianfang Cao, Min Wang, Yanfei Li, Qi Zhang

**Affiliations:** 1 Department of Computer Science & Technology, Xinzhou Teachers University, Xinzhou, China; 2 School of Computer Science & Technology, Taiyuan University of Science and Technology, Taiyuan, China; University of Central Florida (UCF), UNITED STATES

## Abstract

An image classification algorithm based on adaptive feature weight updating is proposed to address the low classification accuracy of the current single-feature classification algorithms and simple multifeature fusion algorithms. The MapReduce parallel programming model on the Hadoop platform is used to perform an adaptive fusion of hue, local binary pattern (LBP) and scale-invariant feature transform (SIFT) features extracted from images to derive optimal combinations of weights. The support vector machine (SVM) classifier is then used to perform parallel training to obtain the optimal SVM classification model, which is then tested. The Pascal VOC 2012, Caltech 256 and SUN databases are adopted to build a massive image library. The speedup, classification accuracy and training time are tested in the experiment, and the results show that a linear growth tendency is present in the speedup of the system in a cluster environment. In consideration of the hardware costs, time, performance and accuracy, the algorithm is superior to mainstream classification algorithms, such as the power mean SVM and convolutional neural network (CNN). As the number and types of images both increase, the classification accuracy rate exceeds 95%. When the number of images reaches 80,000, the training time of the proposed algorithm is only 1/5 that of traditional single-node architecture algorithms. This result reflects the effectiveness of the algorithm, which provides a basis for the effective analysis and processing of image big data.

## Introduction

The selection of image features and classifiers has long been a primary challenge in image classification [1]. Traditional image classification algorithms can be roughly divided into two categories. One category includes classification algorithms that employ artificial markers, whereas the other includes algorithms that use keywords and text to describe and classify images. These methods are simple and easy to understand. However, they are also time consuming, cumbersome and easily affected by subjectivity, and they yield inaccurate classification results [2]. Classification methods [3] based on image contents were subsequently proposed; these methods avoid the effects of subjectivity and display high classification performance. Currently, image classification methods based on low-level visual features are commonly used for image feature extraction, and these methods display both accuracy and temporal complexity advantages in image classification. However, the single-feature descriptions and single-node architecture algorithms used in these methods have considerable defects, such as low classification accuracy and poor time performance when applied to big data [4].

Based on the above ideas, an improved support vector machine (SVM) classification algorithm based on adaptive feature weight updating (AFWU-SVM) is proposed. This algorithm can extract multiple features from images and carry out random adaptive fusion to determine the optimal feature weights for use in fusion. The SVM classification algorithm is adopted to verify the fusion effect. Given the large amount of computational resources required to perform adaptive feature fusion, SVM is a computationally intensive algorithm and exhibits poor performance when used to process large-scale data. To improve the classification efficiency, we apply AFWU-SVM using parallel computing methods on the Hadoop cloud computing platform to achieve a parallel AFWU-SVM (PAFWU-SVM). Experimental verification shows that the proposed algorithm displays high classification accuracy. Furthermore, this method displays shorter run times in addition to higher accuracy and significantly greater efficiency than traditional single-node architecture algorithms while guaranteeing cost effectiveness. Therefore, this method has wide applicability.

## Literature review

In image classification, image characteristics are key factors that determine classification performance. Low-level visual feature algorithms are frequently used for image feature extraction, and image classification methods that are based on single low-level visual features (such as color, texture, or shape) are the most common [5]. Golpour et al. [6] classified 5 rice cultivars according to 36 color features extracted from the RGB, HSI, and HSV spaces of images. In the field of medicine, Sezer et al. [7] extracted the texture features of humeral heads via the Hermite transform and developed a computer-aided diagnosis system to identify images representing normal and edematous humeral heads. Kothari et al. [8] proposed a shape-based image analysis method for renal tumors that used Fourier shape descriptors to extract the shape features from images and captured the distribution of stain-enhanced cellular and tissue structures in each image to distinguish disease subtypes. In addition, other algorithms based on single image features are also commonly used in image classification. Fidalgo et al. [9] classified images in the Caltech101 dataset by extracting the scale-invariant feature transform (SIFT) features from the images using the Edge-SIFT descriptor. Guccione et al. [10] proposed an iterative hyperspectral image classification algorithm based on spectral-spatial relational features by defining the spatial features of remote sensing images. Although researchers have selected different features for use in image classification, single features often fail to accurately describe the contents of images in several application fields. Therefore, the performance of the above single-feature-based image classification algorithms can still be improved. As an alternative, in recent years, many scholars have studied the deeper features of images, that is, the high-level semantic features. For example, Lei et al. [11] applied rough set theory to construct low-level feature decision tables for images and proposed a feature extraction algorithm based on a rough set approach to identify the low-level semantic features of images. In addition, Han et al. [12] proposed an algorithm to extract semantic features from images based on canonical correlation analysis and feature fusion; the application of this algorithm effectively ensured consistency between low-level features and high-level semantics. For remote sensing images, Wang et al. [13] designed an image retrieval scheme suitable for scene matching that combined the visual features of images with the semantic features of object and spatial relationships. However, although semantic features can effectively express the content of an image, semantic features are very complicated, and their extraction is a relatively difficult endeavor. At present, most semantic feature extraction algorithms are based on the low-level visual features of images [14]. Therefore, researchers have recently begun to consider image classification methods that integrate a variety of features. Zakeri et al. [15] extracted the texture and shape features of ultrasound images to differentiate benign and malignant breast masses. Lee et al. [16] proposed an automated detection and segmentation method for computed tomography (CT) images using texture and context feature classification that was intended for use in the computer-aided diagnosis of renal cancer. Dhara et al. [17] described pulmonary nodules in lung CT images using shape-based, margin-based and texture-based features to classify benign and malignant pulmonary nodules to assist in treatment. In the field of agriculture, Dubey et al. [18] studied an apple disease classification method based on color, texture and shape features and experimentally verified the method by comparing the results with individual features. Liu et al. [19] used the local binary pattern (LBP) operator to extract textural features from images and integrated the color information features from the images, improving the efficiency of image classification and retrieval to a certain degree. Mirzapour et al. [20] fused the spectral information contained in images with the texture and shape features of hyperspectral images and performed repeated experiments to determine the optimal combination of features; this practice improved the efficiency of hyperspectral image classification. When applying the multifeature image classification methods described above, it is of great importance to determine the optimal combination of weights of the different features. However, researchers generally determine the weights of various features through repeated experiments or artificial methods. Such approaches are prone to subjectivity and improve the classification effect for only specific kinds of images. The classification effectiveness often decreases when the same features and weights are applied to other types of images. Therefore, identifying a multifeature-weighted algorithm that is not strongly affected by human factors, has low complexity and displays good effectiveness in feature fusion has become a key research topic. Among the various underlying features of different image types, hue features can effectively describe the most common color information contained in images using histograms [21]. Similarly, LBP features possess significant advantages, including gray invariance, and they are simple to calculate [22]. Moreover, SIFT features are characterized by image scaling, rotation and affine transformation invariance, and they exhibit very strong distinguishing capabilities [23]. Thus, these three characteristics are widely used by researchers in the field of computer vision and are suitable for various types of image classification. Accordingly, this paper selects these three features for the fusion and classification of images.

Machine learning constitutes the core of artificial intelligence (AI), and many machine learning methods are currently applied to image classification problems. Su et al. [24] proposed a new feedback algorithm related to image retrieval with learning capabilities based on a Bayesian classifier and using different feedback strategies. Han et al. [25] proposed an ensemble algorithm based on Diverse Ensemble Creation by Oppositional Relabeling of Artificially Training Examples (DECORATEs) and rotation forests to improve the classification of remote sensing images. Hou et al. [26] proposed an adaptive feature-weighted K-nearest-neighbors classification method by analyzing the influence of different features of natural images on the classification results; the experimental results indicated that the proposed algorithm could classify natural images quickly and accurately to meet the user-defined classification precision, time, and complexity requirements. To improve the generalization and learning abilities of SVM-based approaches, Zhao et al. [27] classified remote sensing images using an improved compound kernel function. Bekaddour et al. [28] integrated the k-means, learning vector quantization (LVQ) neural networks and SVM algorithms to classify multispectral satellite images. To solve the problems of overfitting and scarce training data in image classification, Zhang et al. [29] proposed a new learning-free image classification algorithm using a framework that incorporated the naive Bayes nearest neighbor and collaborative representation approaches. Among the common machine learning algorithms mentioned above, SVMs have been widely applied; they have several advantages, such as small sample size requirements, ease of use, and global optimization. However, many studies have not changed the status quo, and SVM algorithms perform poorly when applied to large-scale data. Furthermore, given a rapid increase in the amount of image data, the performance of traditional SVM algorithms declines drastically because they cannot process very large volumes of data. Consequently, in recent years, deep learning has become a new focus of research due to its excellent representation of image features. Deep learning models can autonomously learn features from image data, discard researchers' predesigned features, and then extract the feature patterns from the data according to predesigned feature extraction rules [30]. In 2006, Hinton et al. proposed the concept of layer-by-layer training, and following this proposal, deep learning attracted substantial attention from the academic world. An example of a deep learning model is the convolutional neural network (CNN), which has been widely studied and applied. Researchers have proposed several improved deep learning models for CNNs, such as AlexNet, VGGNet, GoogleNet, and ResNet [31]. In 2012, Krizhevsky et al. trained a CNN using two graphics processing units (GPUs) to classify data in the ImageNet database and achieved a very high classification accuracy [32]. This achievement initiated a deluge of deep learning research in the fields of image classification and target detection. However, because of their relatively high complexity, deep learning models require high computational performance to build, and their complexity constitutes a potential problem with regard to their deployment. Accordingly, the construction of deep learning models requires the use of expensive hardware such as high-end GPUs; therefore, due to their hardware costs and classification performance, deep learning models are not an ideal choice for image classification [33]. Alternatively, the development of cloud computing and related technologies provides a feasible platform for the classification of big data, and these technologies involve the use of data classification algorithms on cloud computing platforms where parallel processing can be conducted. Hadoop is a very simple and distributed open-source computing platform [34] with several very useful properties, such as high efficiency, reliability, fault tolerance, and scalability. The classification performance of many algorithms can be greatly improved by carrying out data classification processing on Hadoop. MapReduce is the core technology of Hadoop, and this software framework is used to process large datasets. The greatest advantage of Hadoop is that it uses parallel computing to process excessively large-scale data, and it allows programs edited with MapReduce to run on clusters composed of many computers with high reliability and fault tolerance. The number of applications that have incorporated MapReduce has increased in recent years [35].

## Methods

### Theoretical background

#### Hue, LBP and SIFT features

The HSV color space [19] is a space in which human perceptions describe colors. HSV directly corresponds to the three elements of the human eye's color visual features: hue (H), saturation (S) and brightness (V). Applying this color space model is suitable for visual judgment by a user. Thus, the HSV color space has become the most common color space. Hue refers to the basic color, such as red, green, blue, and purple. Hue represents the color information of the image, that is, the position of the spectral color in the image, which is a commonly used color feature. Saturation refers to the purity of the color; the saturation of bright red is high, while the saturation of pink is low. The brightness value is the intensity of light. In this paper, the hue component is extracted as one of the features of the image-based HSV space.

LBP [22] was first proposed by T. Ojala, M. Pietikäinen, and D. Harwood in 1994 and is an operator that is used to describe the local texture features of images. LBP has significant advantages, such as rotation invariance and gray invariance and is used to extract texture features from images.

SIFT is a scale-based image feature description operator that was proposed by David G. SIFT is a method for calculating key invariant points such as image position, scale, and rotation by detecting the extreme points from a multiscale image pyramid. SIFT has the characteristics of image scaling, rotation and affine transformation invariance and has strong distinguishing ability; thus, it has become a widely used image feature extraction algorithm. The SIFT calculation process mainly includes constructing the scale space, performing extreme point detection, obtaining scale invariance, filtering and precisely positioning the feature points, assigning direction values to each feature point, and generating feature descriptors.

Since the above features are the three most commonly used in automatic image classification, the image content can be described from invariant key points such as color, texture, image position, scale, and rotation, which more fully express the image's implied content. Therefore, this paper uses the above three features as the feature inputs for the SVM classifier in repeated experiments.

#### SVM classification algorithm

The SVM algorithm was derived from statistical learning theory. The algorithm is based on the structural risk minimization principle; it can compress the collection of raw data to a support vector set (usually 3–5% of the raw data) and learn to obtain a classification decision function. The basic idea is to construct a hyperplane as the decision surface so that the interval between the positive and negative mode is maximum.

**Fig 1:**
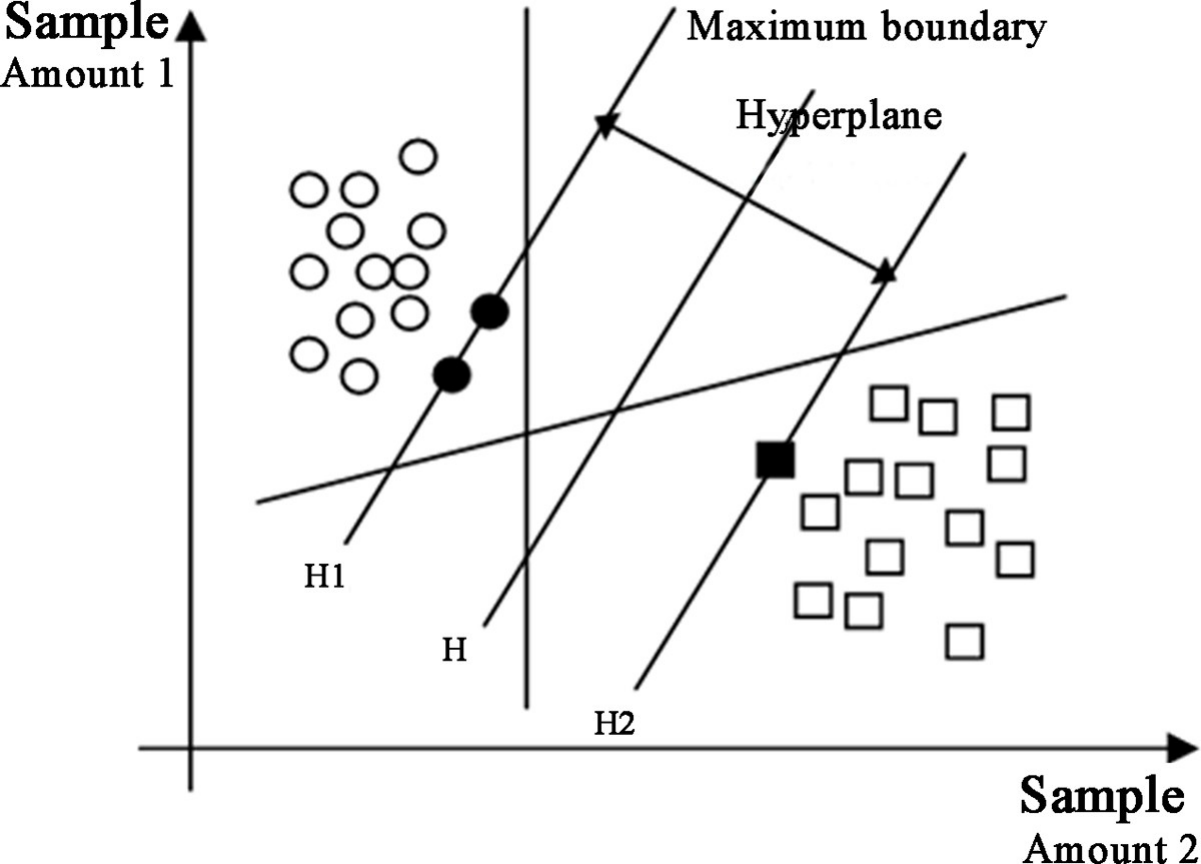
The optimal classification surface.

The SVM method is linearly separable from the optimal classification surface (Fig 1).

The hollow circles and open squares represent the two types of training samples. *H* is the classification line that is correctly separated. *H1* and *H2* not only pass through the points that are nearest to the various sample types but also are parallel to the classification line, and the distance between the two lines is called the classification interval. In accordance with the principle of empirical risk minimization theory, the actual SVM risk is decided by the formula *R*(*ω*)≤*R*_*emp*_(*ω*)+Φ, where *R*(*ω*) represents the actual risk, *R*_*emp*_(*ω*) represents the empirical risk, and Φ represents the confidence interval. Complete separation ensures *R*_*emp*_(*ω*) = 0, and a maximum interval ensures the minimum range of the confidence interval Φ so that the true risk is minimized.

#### AFWU-SVM algorithm

**Fig 2:**
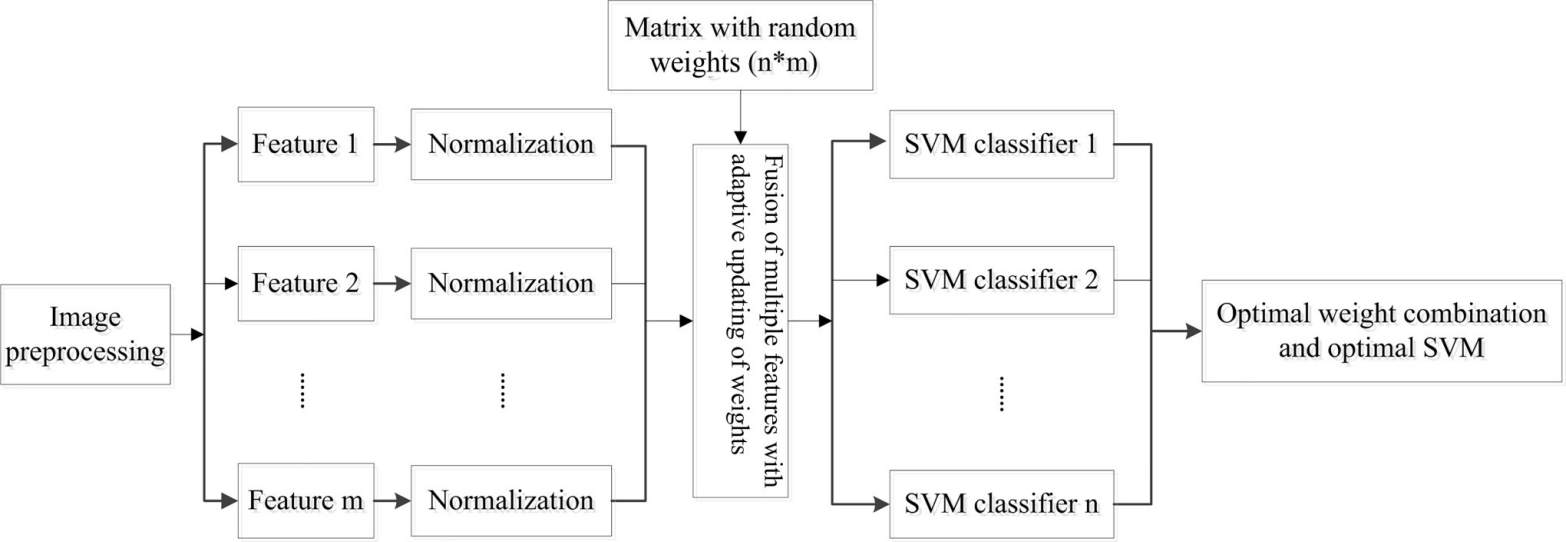
Framework of the AFWU-SVM algorithm.

The framework of the AFWU-SVM algorithm proposed in this study is shown in Fig 2. The algorithm consists of six steps, namely, feature extraction, feature normalization, the production of a matrix with random weights, feature fusion, classifier training, determination of the optimal combination of weights and identification of the corresponding SVM classifiers. This algorithm changes a single feature to a fusion feature using a matrix with random weights, and it then trains multiple classifiers to further obtain the optimal combination of weights and the optimal SVM classifier. The feature extraction stage and the SVM training stage of this algorithm are mutually independent, satisfying the implementation conditions of parallel algorithms. Therefore, this technology can be applied to big data to improve computational efficiency.

#### Feature normalization

Eigenvectors differ in terms of their dimensions, and they also vary strongly in terms of their values. When fused into one eigenvector, they need to be processed according to certain rules [36]. The numerical values of different features extracted from the same image differ strongly. To avoid the influence of this numerical disparity on the classification results, this study uses the linear normalization method [37] to scale the feature values to a designated range to reduce the impact of the different ranges of values on the importance of the various eigenvectors. Suppose that the original eigenvectors are {*f*_1_,*f*_2_,⋯,*f*_*m*_}, max(*f*) is the maximum value of {*f*_1_,*f*_2_,⋯,*f*_*m*_}, min(*f*) is the minimum value of {*f*_1_,*f*_2_,⋯,*f*_*m*_}, and $\left\{f_{1}^{'},f_{2}^{'},\cdots ,f_{m}^{'}\right\}$ are the normalized eigenvectors. The extracted image features are normalized according to Eq (1) below.

$$f_{i}^{'}=\{\begin{array}{c} 1\;f_{i}=\max(f)\\ 0\;f_{i}=\min(f)\\ \frac{f_{i}}{\max(f)-\min(f)}\;others \end{array}\;i=1,2,\cdots ,m$$ (1)

#### Feature fusion rules

Some relevant mathematical descriptions are given first to describe the AFWU-SVM algorithm.

(1) Matrix with random weights

Suppose that $\left[\begin{array}{cccc} w_{11} & w_{12} & \cdots & w_{1m}\\ w_{21} & w_{22} & \cdots & w_{2m}\\ \vdots & \vdots & \vdots & \vdots \\ w_{n1} & w_{n2} & \cdots & w_{nm} \end{array}\right]$ is a matrix *W*_*n*×*m*_ with random weights and a rank of *R*(*W*_*n*×1_) = *n*, *w*_*ij*_ is a randomly generated element in *W*_*n*×*m*_, and 0<*w*_*ij*_<1. When *w*_*i*1_+*w*_*i*2_+⋯+*w*_*im*_ = 1(*i* = 1,2,…,*n*), a set of random weights (*w*_*i*1_,*w*_*i*2_,⋯,*w*_*im*_) is generated. N groups of random weights constitute the matrix with random weights.

(2) Fusion eigenvector matrix

Suppose that $\left\{f_{1}^{'},f_{2}^{'},\cdots ,f_{m}^{'}\right\}$ are the normalized eigenvectors. The fusion eigenvector matrix *F*_*n*×1_ can be derived according to Eq (2). Each row of the matrix represents a fusion eigenvector, and the size of *n* depends on the number of rows in the random weight matrix.

$$F_{n\times 1}=\left[\begin{array}{c} w_{11}\cdot f_{1}^{'}+w_{12}\cdot f_{2}^{'}+\cdots +w_{1m}\cdot f_{m}^{'}\\ w_{21}\cdot f_{1}^{'}+w_{22}\cdot f_{2}^{'}+\cdots +w_{2m}\cdot f_{m}^{'}\\ \vdots \\ w_{n1}\cdot f_{1}^{'}+w_{n2}\cdot f_{2}^{'}+\cdots +w_{nm}\cdot f_{m}^{'} \end{array}\right]=W_{n\times m}\times \left[\begin{array}{c} f_{1}^{'}\\ f_{2}^{'}\\ \vdots \\ f_{m}^{'} \end{array}\right]$$ (2)

Finally, the feature fusion rule *C*_*i*_ for the *i*th SVM classifier is expressed by Eq (3):

$$C_{i}=w_{i1}\cdot f_{1}^{'}+w_{i2}\cdot f_{2}^{'}+\cdots +w_{im}\cdot f_{m}^{'},\;w_{i1}+w_{i2}+\cdots +w_{im}=1$$ (3)

#### Feature fusion and classification method

After the AFWU-SVM algorithm normalizes the features of each image where the data converge, Eq (3) is then used to derive the datasets comprised of fusion eigenvectors. The data are divided into a training dataset and a testing dataset at a ratio of 2:1. The former is used to train the classification model, while the latter is used to determine the classification accuracy of this model. In this way, *n* groups of random weights are used to train *n* SVM classifiers, and a classification model set *A* = {*C*_1_,*C*_2_,⋯,*C*_*n*_} and a classification accuracy set *B* = {*T*_1_,*T*_2_,⋯,*T*_*n*_} are then obtained. The data in set *B* are sorted in descending order to obtain the optimal combination of weights (*w*_*x*1_,*w*_*x*2_,⋯,*w*_*xm*_) and the corresponding optimal SVM model *C*_*x*_.

### Design and implementation of the parallel AFWU-SVM (PAFWU-SVM) algorithm

#### Parallel platform and programming model

**Fig 3:**
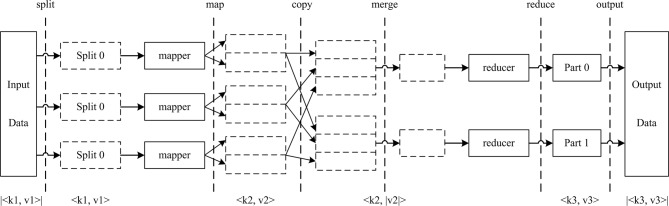
Processing flow of a task using the MapReduce model. k, the key of the key-value pairs; v, the value of the key-value pairs.

Hadoop [38] is a distributed system infrastructure that was developed by the Apache Foundation, and it is well recognized as a platform for handling massive amounts of data and addressing complex computing problems. The Hadoop Distributed File System (HDFS) and MapReduce are two core elements of the Hadoop platform. The HDFS enables streaming access to large datasets by adopting a host-guest architecture. MapReduce is a parallel programming model that can assign computing tasks and data to each node of a Hadoop cluster. By means of functional programming methods, the computing is divided into two processes, i.e., Map and Reduce. Key-value pairs are handled as inputs and outputs for each process. By defining a Mapper class and a Reduce class, one key-value pair is mapped to another key-value pair. The processing flow is shown in Fig 3 [39].

### PAFWU-SVM

(1) Overall framework of the algorithm

**Fig 4:**
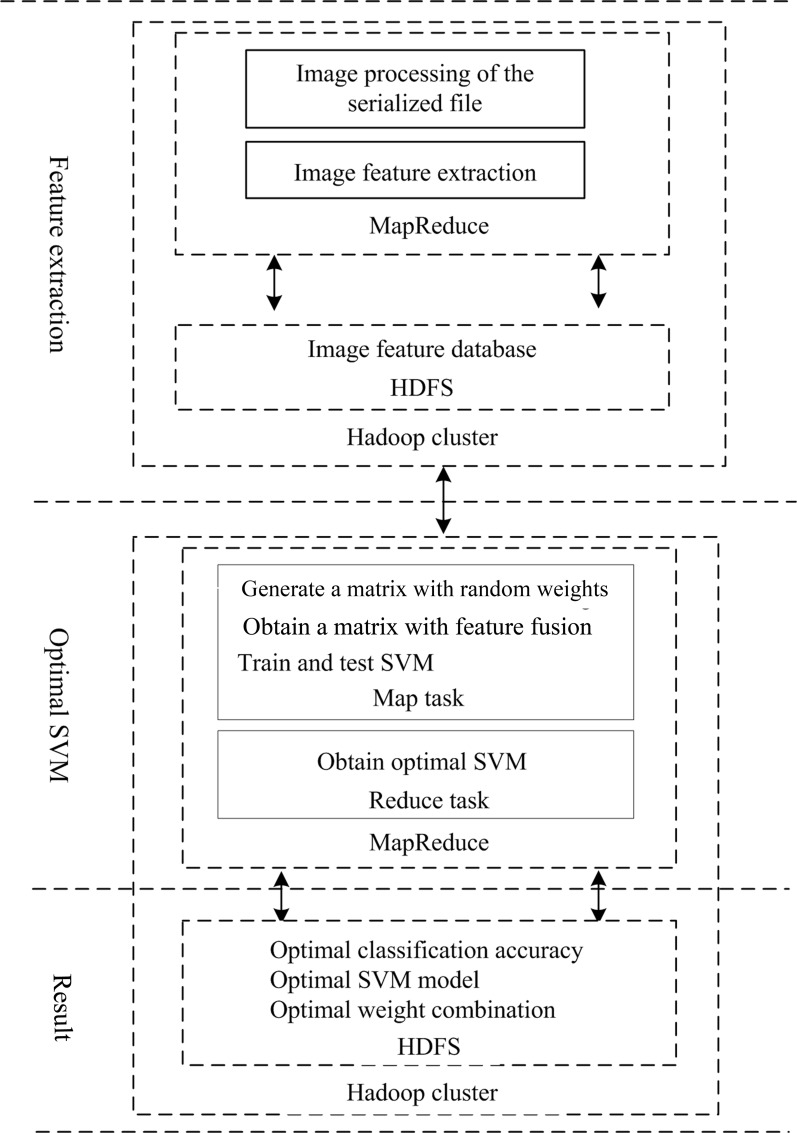
Overall framework of the PAFWU-SVM algorithm.

The overall framework of the AFWU-SVM algorithm designed using the MapReduce parallel programming model and implemented on the Hadoop platform is shown in Fig 4.

The algorithm as a whole is divided into two parts. The first part comprises the extraction of image features; in this study, three types of features, namely, hue, LBP and SIFT features, are extracted. The second part is composed of three steps: feature fusion, obtaining the optimal combination of weights and determining the corresponding classification model. In the MapReduce parallel programming model, the Map task and the Reduce task mainly consist of the setup(), mapper(), reducer(), and cleanup() functions.

(2) Algorithm design and implementation

OpenCV is an open-source and cross-platform computer vision library that contains many algorithms that are used in digital image processing and provides a large number of Java interfaces [40]. Thus, this study uses the OpenCV function library to extract and classify the image features. Moreover, the principal component analysis (PCA) algorithm, which can purify the eigenvectors by removing noise and improving the matching rate, is used to reduce the dimensionality of the SIFT features [41]. Java programming is used to implement the PAFWU-SVM algorithm on the Hadoop platform. In addition, because the parameter values used in the SVM have a strong impact on the classification results, K-fold cross validation is used to identify the best parameters of the radial basis function. The results of repeated experiments verify that the parameters obtained are most suitable when K is equal to 10. The algorithm is described as follows:

*First stage*: feature extraction

Step 1. The small image files in the input dataset are processed into a serialized file and saved in the HDFS file system.
Step 2. The mapper() function reads the serialized file and processes the images to a uniform 150*200 size.
Step 3. The mapper() function obtains information on the category of each image. This function also extracts the hue, LBP and SIFT features of the corresponding images and normalizes these features.
Step 4. The category information and features corresponding to each image are organized into <category, feature value> key-value pairs and saved in the HDFS.

*Second stage*: image classification

//Map task:

- Step 1. The setup() function generates the random weight matrix *W*_*n*×3_.
- Step 2. The eigenvector set *WF* after feature fusion is obtained according to Eq (3).
- Step 3. *WF* is randomly divided into the training set *WF*_1_ and testing set *WF*_2_ at a ratio of 2:1.
- Step 4. Each element in *WF*_1_ is fed into the SVM to obtain the trained classification model *C*_*i*_.
- Step 5. Each element in *WF*_2_ is fed into the trained classification model to derive the classification accuracy *T*_*i*_ of the corresponding model.
- Step 6. Steps 2–5 are executed repeatedly to obtain the set of classification models *A* = {*C*_1_,*C*_2_,⋯,*C*_*n*_} and the set of classification accuracy values *B* = {*T*_1_,*T*_2_,⋯,*T*_*n*_}.

//Reduce task:

- Step 7. The reducer() function sequences the data in the set of classification accuracy values *B* obtained in Step 6 to determine the optimal combination of weights and the optimal SVM classification model.
- Step 8. The optimal combination of weights and their corresponding model paths are fed into the HDFS.

The source code of the algorithm is provided in the S1 File.

### Experimental design

#### Experimental environment

In this study, a Hadoop cluster is built using five computers, and the TensorFlow framework was built on the Hadoop platform. One of the machines is the master node, and the remaining 4 computers are slave nodes. All of the node computers include an Intel i7 four-core eight-thread 4.2 GHz processor with 8 GB of memory and a 4 TB hard disk as well as the following software: version 14.04 of the 64-bit Ubuntu operating system, the jdk1.7.0_79 Java environment, and Hadoop-2.5.1 (64-bit compiled).

#### Experimental data

The experimental data used in this study originate from free public datasets containing images from different categories: the Pascal visual object class (VOC) 2012 image database (17,125 images, 20 categories), the Caltech 256 image database (30,607 images, 256 categories) and the SUN database (131,067 images, 908 categories). In this paper, 80,000 images including 635 categories are randomly selected from the three databases for experimentation: 10,000 images including 15 categories are chosen from the Pascal VOC 2012 image database, 20,000 images including 193 categories are selected from the Caltech 256 image database, and 50,000 images including 427 categories are obtained from the SUN database. The first 65% of the images in each category are used as the training dataset, and the remaining 35% of the images are used as the testing dataset.

ImageNet is a large image database often used for research on visual object recognition. The dataset includes more than 14 million images, covers more than 20,000 categories and is widely used in the deep learning field. Studies on image classification, positioning and detection are mostly based on the images in this dataset. To further verify the applicability and universality of the proposed algorithm, we randomly selected 5 million images from 8,350 categories from the ImageNet database and compared the training time and classification accuracy of the proposed algorithm with those of other commonly used deep learning algorithms.

## Results and discussion

This study conducts a comparison experiment that considers three aspects, namely, the system speedup, classification accuracy and training time.

### System speedup

**Fig 5:**
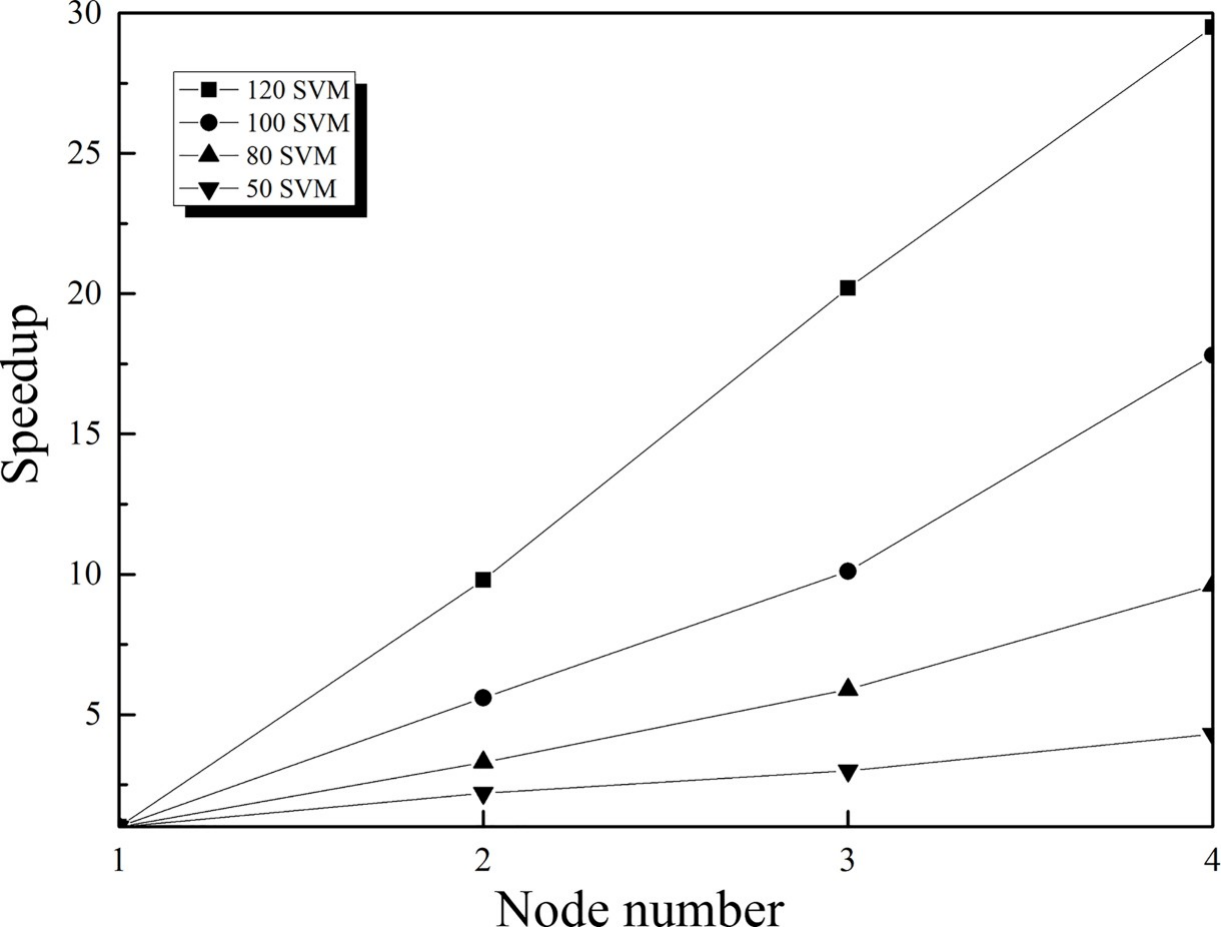
Comparison of the speedup values.

Speedup [42] refers to the ratio of the run time of a task in a single-node environment to the run time of the same task in a multinode environment. Speedup is an important index that is used to measure the efficiency of the parallel algorithm on the Hadoop platform. To verify the performance of the proposed algorithm on the Hadoop platform, this study conducts a speedup experiment by training 50, 80, 100, and 120 SVMs. The results of the system speedup experiment are shown in Fig 5.

In an ideal case, the speedup of a system will increase linearly with the increase in the number of node computers. However, due to communication overhead and load balance, the speedup does not increase linearly in practice. Fig 5 shows that the speedup of the system increases with the increase in the number of node computers when the number of trained SVMs is small, although the growth rate is moderate. However, the speedup of the system increases with the increase in the number of trained SVMs. When the number of trained SVMs reaches 120, the speedup of the system shows a nearly linear growth tendency, which further demonstrates that the Hadoop cluster is superior in dealing with large amounts of complex computations.

### Classification accuracy

To verify the effectiveness of the proposed algorithm, this study first conducts experiments involving 20,000 images to compare the classification accuracy obtained via single-feature classification with that obtained via multifeature fusion at a ratio of 1:1:1 and that of the optimal SVM identified by random fusion training. To verify the effects of the feature fusion method and classification algorithm proposed in this paper, we use different classification algorithms to compare the classification effects of different feature fusion methods on different datasets. The results are shown in Table 1.

**Table 1:** Comparison of the classification accuracies of different algorithms and different feature fusion methods.

Algorithms	Extracted features and the weight ratio among the features for fusion	Classification accuracy				
		Database in the literature [10] (320 images)	Pascal VOC 2012 (10,000 images)	Caltech 256 (20,000 images)	SUN database (50,000 images)	Average classification accuracy
MSVM [18]	Color	86.42%	78.45%	70.18%	56.49%	71.05%
	SIFT	88.68%	79.29%	72.21%	59.02%	74.13%
	The method in the literature [18] Color:LBP:Shape = 1:1:1	95.94%	85.41%	77.94%	60.50%	81.26%%
	Hue:LBP:SIFT = 1:1:1	95.95%	86.49%	79.26%	63.77%	82.93%
PMSVM [43]	Color	88.43%	84.66%	82.15%	81.06%	83.92%
	SIFT	91.07%	88.37%	86.43%	84.81%	87.49%
	The method in the literature [18] Color:LBP:Shape = 1:1:1	96.89%	92.24%	91.03%	87.16%	90.42%
	Hue:LBP:SIFT = 1:1:1	96.91%	94.01%	92.86%	89.41%	94.10%%
PSVM-KNN [44]	Color	88.92%	85.04%	82.19%	81.89%	85.23%
	SIFT	91.46%	89.02%	87.33%	86.25%	89.07%
	The method in the literature [18] Color:LBP:Shape = 1:1:1	96.90%	92.56%	91.48%	89.07%	91.41%
	Hue:LBP:SIFT = 1:1:1	97.02%	94.22%	93.56%	91.73%	95.01%
The proposed method	Color	86.14%	83.27%	82.42%	81.00%	82.96%
	SIFT	86.99%	84.55%	84.97%	83.71%	85.15%
	Color, LBP, Shape feature adaptive fusion	98.78%	93.26%	92.81%	91.39%	94.18%
	The method in this paper: hue, LBP, SIFT feature adaptive fusion	99.02%	96.81%	96.32%	95.39%	97.03%

Notes: Color:LBP:Shape = 1:1:1 and Hue:LBP:SIFT = 1:1:1 mean that the three extracted features are fused at a 1:1:1 ratio for classification. In the algorithm proposed in this study, the extracted features are fused according to the adaptive feature weight updating method.

The results in Table 1 clearly reveal that the method proposed in this paper exhibits a far superior classification performance with regard to the Hue, LBP and SIFT features than the methods used to fuse color, LBP and shape features reported in the literature [18]. The adaptively weighted feature fusion method proposed in this paper has a higher classification accuracy than the common 1:1:1 feature fusion method and the classification methods that extract a single feature (Color, SIFT). Among the classification algorithms listed in Table 1, the methods that extract a single feature (Color, SIFT) for image classification exhibit obviously lower accuracy than the classification method that utilizes feature fusion. However, for the experimental image library used in this paper, after adaptively merging the Hue, LBP and SIFT features, the classification performance is significantly better than that of the extracted Color, LBP and Shape features in the literature [18] for 1:1:1 fusion and adaptive fusion. In addition, Dubey and Jalal used the single-node architecture of the multicategory SVM (MSVM) algorithm [18] and found that the classification accuracy decreased significantly with the increase in the size of the database. In contrast, the methods used by Wang et al. [43] and Cao et al. [44] and the algorithm proposed in this study are all based on the Hadoop cluster architecture, and thus, their classification accuracies do not drastically decrease with a dramatic increase in the number of images. As a consequence of the enhancement attributable to the distributed computing power, the classification accuracies of the methods based on this architecture are significantly higher than those of the methods in the literature [18] based on the single-node architecture (e.g., the MSVM algorithm). Accordingly, the proposed method is suitable for the classification of multiple categories of images because it is optimized for training in a cluster environment; therefore, its classification performance is optimal relative to other algorithms. Additionally, when the number of image datasets is small, the algorithms with a single-node architecture and multinode architecture in a cluster environment do not exhibit notably different performance; however, when the size of the image dataset increases, the difference will become increasingly obvious, thereby illustrating the powerful distributed computing power of the Hadoop cluster environment.

**Fig 6:**
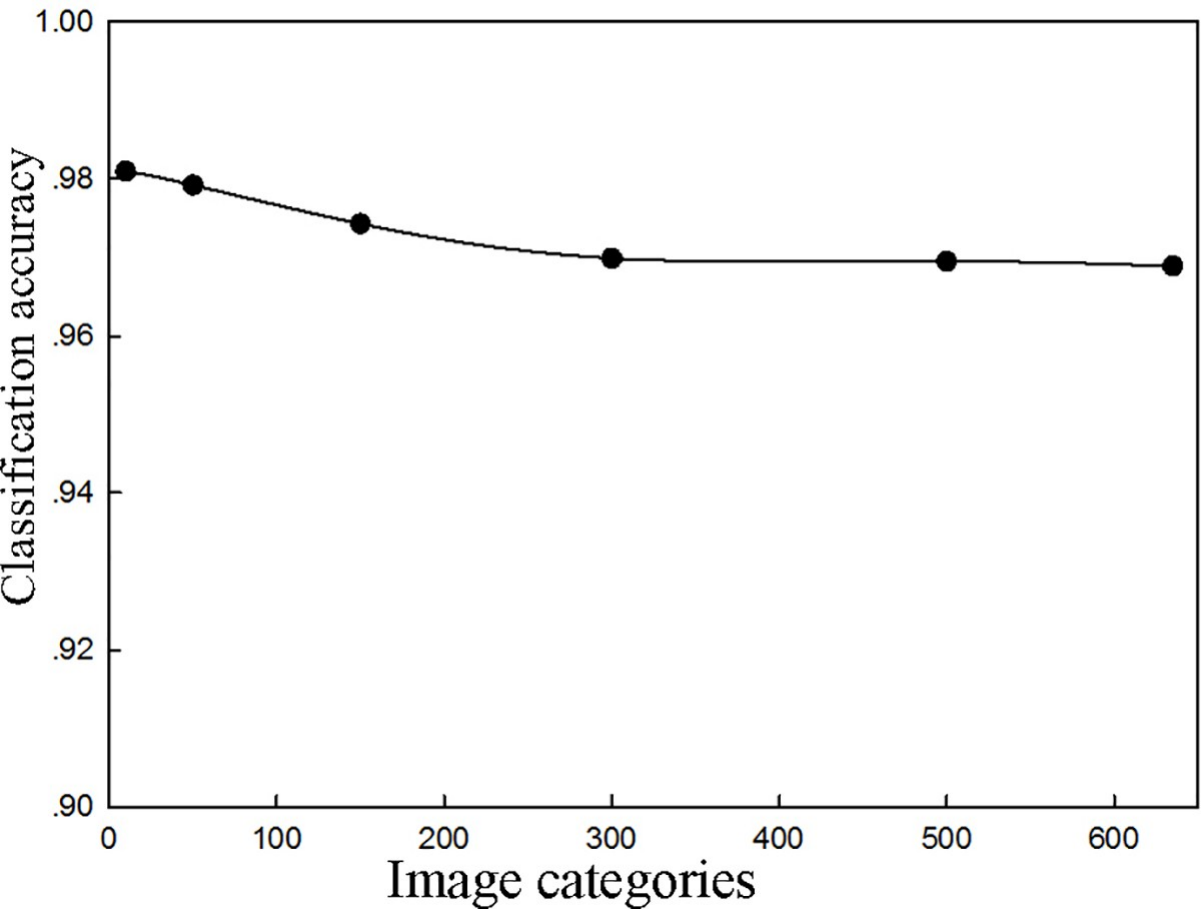
Comparison of the average classification accuracies of the proposed algorithm for different numbers of classification categories with varying numbers of images (Notes: 10 categories: 1,200 images; 50 categories: 6,000 images; 150 categories: 16,500 images; 300 categories: 31,800 images; 500 categories: 51,095 images; 635 categories: 80,000 images).

Furthermore, to verify the stability of the proposed method, we also compare the relationship between the number of image categories and the accuracy; the results are shown in Fig 6.

Fig 6 clearly demonstrates that the classification accuracy of the proposed method remains above 96.5% without an obvious downward trend, even though the number of classification categories and number of images are both constantly increasing. This result indicates that the proposed adaptively weighted feature fusion classification method is stable in the cluster environment, and its classification performance does not exhibit large fluctuations with sharp increases in the numbers of either images or categories.

### Training time

**Fig 7:**
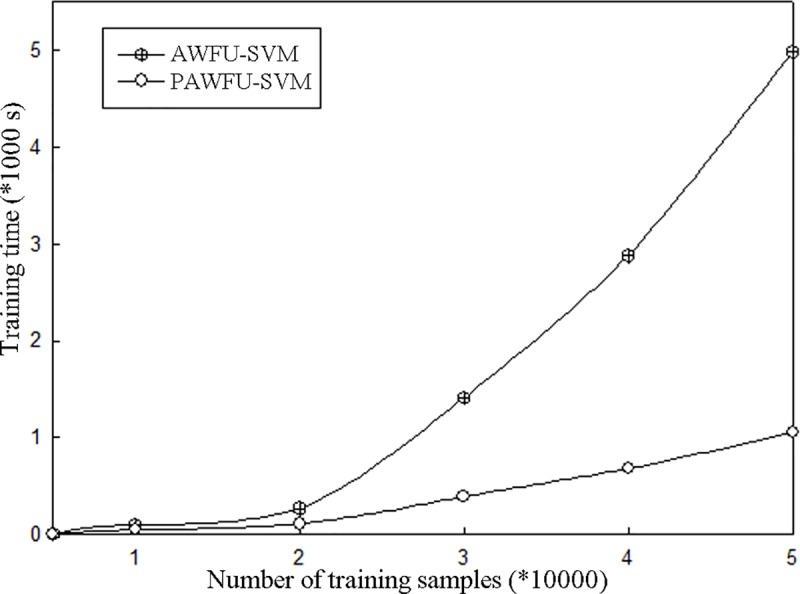
Comparison of the training times among different algorithms.

To further verify the effectiveness of the parallel algorithm in the cluster environment in terms of its time performance when applied to training samples of different scales, this study conducts experiments to compare the training time of the proposed image classification method based on adaptive feature weight updating in a single-node architecture environment with that in the cluster environment. A comparison of the resulting training times is shown in Fig 7.

Fig 7 shows that the training times of the PAFWU-SVM algorithm in the cluster environment and the AFWU-SVM algorithm under the traditional single-node architecture do not differ greatly for relatively small training samples. However, as the size of the training data sample increases, the training time of the AFWU-SVM algorithm under the single-node architecture shows an exponentially increasing tendency. When the training dataset contains the same number of samples, the training time of the AFWU-SVM algorithm is far longer than that of the PAFWU-SVM algorithm based on the MapReduce parallel programming model. Moreover, as the number of training samples increases, the increase in training time becomes more obvious, which fully indicates that distributed parallel computing methods can be used in cluster environments to analyze big data.

In addition, to better verify the comprehensive performance of the proposed method in terms of the training time and classification accuracy, we combine these indices with the currently popular deep learning algorithms (CNN, AlexNet, VGGNet, GoogleNet, ResNet and DenseNet) [31] for experimental comparison, and the results are shown in Table 2.

**Table 2:** Comprehensive comparison between the algorithm proposed in this paper and other deep learning algorithms.

Number of images	Index	Method						
		CNN	AlexNet	VGGNet	GoogleNet	ResNet	DenseNet	Method of this paper
5,000	Training time (S)	604	621	619	635	640	643	1.2
	Classification accuracy (%)	97.93	97.92	97.93	97.93	97.92	97.93	97.92
10,000	Training time (S)	1080	1090	1085	1120	1103	1032	50
	Classification accuracy (%)	96.81	96.81	96.82	96.83	96.83	96.84	96.83
25,000	Training time (S)	2700	2702	2765	2748	2801	2792	296
	Classification accuracy (%)	96.18	96.17	96.18	96.18	96.18	96.18	96.17
50,000	Training time (S)	5310	5401	5400	5376	5412	5400	1050
	Classification accuracy (%)	95.27	95.27	95.27	95.28	95.30	95.30	95.28
80,000	Training time (S)	10245	10283	10300	10299	10307	10308	1458
	Classification accuracy (%)	95.15	95.15	95.16	95.15	95.16	95.18	95.14

Notes: Due to the experimental conditions, a GPU is expensive. This article uses deep learning algorithms for experimentation in the CPU environment based on the TensorFlow framework.

The results in Table 2 show that both the deep learning algorithms and the method proposed in this paper have nearly identical classification accuracies. However, due to the complexity of the training process, the deep learning algorithms run on the CPU over a long training time (usually tens of minutes or more). Although this article builds the TensorFlow deep learning framework on the Hadoop platform, the training time is still very long due to the CPU. In fact, the deep learning algorithms need to be supported by a GPU, which would greatly shorten the training time. However, the cost of a GPU is high (in China, the cost of a GPU is currently approximately ten times that of a CPU), and it is difficult for general laboratories and scientific research institutions to establish an environment with the appropriate hardware required by the deep learning algorithms. Therefore, under the premise of comprehensively weighing the hardware cost and classification performance, the algorithm proposed in this paper can achieve almost the same classification accuracy rate as the deep learning algorithm with a short training time, which greatly saves hardware costs, and the experimental effect is ideal. The training time and classification performance of the proposed method in this paper are improved.

To better verify the applicability of the proposed method, we also conducted experimental comparisons of training time and classification accuracy on the ImageNet dataset and obtained similar results (the outcomes of these analyses are shown in the S2 File).

## Conclusions

This study conducts a detailed investigation of the multi-image feature adaptive fusion method and the SVM classification algorithm on the Hadoop platform and applies the MapReduce parallel programming model to the proposed algorithm to solve the issues associated with its high computational burden, poor time performance and low classification accuracy. While guaranteeing its time performance, the classification accuracy of the algorithm is effectively improved.

The experimental results show that the proposed algorithm features high accuracy, requires comparatively little time and is not strongly influenced by human factors. The Hadoop cluster built in this study makes full use of the resources of the node computers and obtains good speedup; it fully embodies the powerful computing capacity of the distributed parallel processing of the Hadoop cluster compared with single-node computers. In consideration of hardware costs, the algorithm proposed in this study solves the problem of the efficient classification of massive image data and describes a novel concept for implementation in the analysis and processing of big data. Compared with the CNN algorithm, the proposed algorithm reduces training time and hardware costs while achieving the expected effect and similar accuracy levels. In addition, this study lays the foundation for the processing of big data in other fields.

However, although the method proposed in this paper is superior to other deep learning methods in terms of time performance, and the classification accuracy is almost the same as that of deep learning algorithms, the time performance problem can be solved by increasing the hardware and software costs. This paper is based on a method that does not increase the costs of hardware and software. In fact, the costs of hardware and software can be gradually addressed in scientific research, which is a limitation of this paper. At present, an important challenge in AI studies is enabling computers to recognize and classify massive images as done by human beings and achieve human-machine interaction. The eventual goal of image classification is to endow computers with the ability to think logically and make judgments matching those of human beings. Given the advent of the era of big data, the analysis and processing of image big data have become a new key topic of research. Based on this study, the possible future research directions are as follows:

1. The content contained in images is very rich. Therefore, how to standardize the classification of image categories more reasonably deserves further in-depth study.
2. The multi-image feature adaptively weighted fusion method should be further studied. The best feature weight combination must be ensured while reducing the amount of computation.
3. Nodes should be added, a distributed deep learning framework should be built, the deep learning method should be utilized to classify massive images, and classification time performance and classification accuracy should be improved.

## Supporting information

S1 File The source codes of the algorithm used in this study. (DOC)

S2 File Training time and classification accuracy comparisons of different algorithms on the ImageNet dataset. (DOC)

## References

[1] Nanni L, Lumini A, Brahnam S. Survey on LBP based texture descriptors for image classification. Expert Syst Appl. 2012;39: 3634–3641.

[2] Du PJ, Xia JS, Zhang W, Tan K, Liu Y, Liu SC. Multiple classifier system for remote sensing image classification: A review. Sensors 2012;12: 4764–4792. 10.3390/s120404764 22666057 PMC3355439

[3] Zhu S, Sun X, Jin D. Multi-view semi-supervised learning for image classification. Neurocomputing 2016;208: 136–142.

[4] Fernando B, Fromont E, Tuytelaars T. Mining mid-level features for image classification. Int J Comput Vis. 2014;108: 186–203.

[5] Banerji S, Sinha A, Liu C. New image descriptors based on color, texture, shape, and wavelets for object and scene image classification. Neurocomputing 2013;117: 173–185.

[6] Golpour I, Parian JA, Chayjan RA. Identification and classification of bulk paddy, brown, and white rice cultivars with colour features extraction using image analysis and neural network. CZECH J Food Sci. 2014;32: 280–287.

[7] Sezer A, Sezer HB, Albayrak S. Hermite-based texture feature extraction for classification of humeral head in proton density-weighted MR images. Neural Comput Appl. 2017;28: 3021–3033.

[8] Kothari S, Phan JH, Young AN. Histological image classification using biologically interpretable shape-based features. BMC Med Imaging. 2013;13: 9 10.1186/1471-2342-13-9 23497380 PMC3623732

[9] Fidalgo E, Alegre E, Gonzalez-Castro V, Fernández-Robles L. Compass radius estimation for improved image classification using Edge-SIFT. Neurocomputing 2016;197: 119–135.

[10] Guccione P, Mascolo L, Appice A. Iterative hyperspectral image classification using spectral-spatial relational features. IEEE Transactions on Geoscience and Remote Sensing, 2015;53: 3615–3627.

[11] Lei S, Gu Y, Cao C, Xie G. Image low-level semantic feature extraction based on rough set. IEEE International Conference on Computer Science and Automation Engineering 2012; 3: 680–683.

[12] Han CG, Guo YT. CCA-based analysis and research of image semantic feature extraction. Application Research of Computers 2012; 29: 1938–1942.

[13] Wang M, Wan QM, Gu LB, Song TY. Remote-sensing image retrieval by combining image visual and semantic features. International Journal of Remote Sensing 2013; 34: 4200–4223.

[14] Fauzi F, Belkhatir M. Image understanding and the web: a state-of-the-art review. Journal of Intelligent Information Systems 2014; 43: 271–306.

[15] Zakeri FS, Behnam H, Ahmadinejad N. Classification of benign and malignant breast masses based on shape and texture features in sonography images. J Med Syst. 2012;36: 1621–1627. 10.1007/s10916-010-9624-7 21082222

[16] Lee HS, Hong H, Kim J. Detection and segmentation of small renal masses in contrast-enhanced CT images using texture and context feature classification. IEEE 14TH International Symposium on Biomedical Imaging (ISBI 2017), pp. 583–586, Melbourne, Australia, APR 18–21, 2017.

[17] Dhara AK, Mukhopadhyay S, Dutta A, Garg M, Khandelwal N. A combination of shape and texture features for classification of pulmonary nodules in lung CT images. J Digital Imaging. 2016;29: 466–475. 10.1007/s10278-015-9857-6 PMC4942385 26738871

[18] Dubey SR, Jalal AS. Apple disease classification using color, texture and shape features from images. Signal Image and Video Processing 2016;10: 819–826.

[19] Liu PZ, Guo JM, Chamnongthai K. Fusion of color histogram and LBP-based features for texture image retrieval and classification. Inform Sci. 2017;30: 95–111.

[20] Mirzapour F, Ghassemian H. Improving hyperspectral image classification by combining spectral, texture, and shape features. Inter J Remote Sensing. 2015;36: 1070–1096.

[21] Yan J. Hue-based feature detection for geometry calibration of multiprojector arrays. Optical Engineering 2014;53: 063108.

[22] Liu P, Guo JM, Chamnongthai K, Prasetyo H. Fusion of color histogram and LBP-based features for texture image retrieval and classification. Information Sciences 2017:390: 95–111.

[23] David G. Distinctive image features fromscal-invariant keypoints, International Journal in Computer Vision. 2004; 60: 91–110.

[24] Su Z, Zhang HJ, Ma SP. An image retrieval relevance feedback algorithm based on the Bayesian Classifier. J Software. 2002;13: 002001–2006.

[25] Han M, Zhu X, Yao W. Remote sensing image classification based on neural network ensemble algorithm. Neurocomputing 2012;78: 133–138.

[26] Hou YT, Peng JY, Hao LW, Wang R. Research of classification method for natural images based on adaptive feature-weighted K-nearest neighbors. Appl Res Comput. 2014;31: 957–960.

[27] Zhao J, Gao W, Liu ZL, Mou GF, Lu L, Yu LN. A classification of remote sensing image based on improved compound kernels of SVM. Russian Journal of Inorganic Chemistry 2010;53: 594–597.

[28] Bekaddour A, Bessaid A, Bendimerad FT. Multi spectral satellite image ensembles classification combining k-means, LVQ and SVM classification techniques. Journal of the Indian Society of Remote Sensing 2015;43: 671–686.

[29] Zhang X, Hao S, Xu C, Qian X, Wang M, Jiang J. Image classification based on low-rank matrix recovery and Naive Bayes collaborative representation. Neurocomputing 2015;169: 110–118.

[30] Zeiler MD, Fergus R. Visualizing and understanding convolutional networks. New York: Springer International Publishing 2013; 818–833.

[31] Zhang QC, Yang LT, Chen ZK, et al A survey on deep learning for big data. Information Fusion 2018; 42: 146–157.

[32] Krizhevsky A, Sutskever I, Hinton G E. Imagenet classification with deep convolutional neural networks. In: Proceedings of the 25th International Conference on Neural Information Processing Systems. Lake Tahoe, Nevada, USA: Curran Associates. 2012; 1097–1105.

[33] Guo Y, Liu Y, Oerlemans A, Lao S, Wu S, Lew MS. Deep learning for visual understanding: A review. Neurocomputing. 2016; 187: 27–48.

[34] White T, Cutting D. Hadoop: the definitive guide. O’reilly Media Inc Gravenstein Highway North; 2012;215: 1–4.

[35] Bechini A, Marcelloni F, Segatori A. A MapReduce solution for associative classification of big data. Inform Sci. 2016;332: 33–55.

[36] Yuan GL, Xue MG, Han YS, Zhou PC. Mean shift object tracking based on adaptive multi-features fusion, J Comput Res Develop. 2010;47: 1663–1671.

[37] Wu Y, Li L. Sample normalization methods in quantitative metabolomics. J Chromatography A. 2016;1430: 80–95. 10.1016/j.chroma.2015.12.007 26763302

[38] White T. Hadoop: The Definitive Guide, 3rd edition California. USA: O’reilly Media; 2012.

[39] Bersani MM, Bianculli D, Ghezzi C, Pietro PS. Efficient large-scale trace checking using MapReduce. IEEE/ACM International Conference on Software Engineering 2017;2017: 888–898.

[40] Aljasem DK, Heeney M, Gritti AP, Raimondi F. On-the-Fly Image Classification to Help Blind People, International Conference on Intelligent Environments, London, UK: IEEE, pp. 155–158, 2016.

[41] Guo Y, Bennamoun M, Sohel F, Lu M. A comprehensive performance evaluation of 3D local feature descriptors. Inter J Comput Vis. 2016;116:. 66–89.

[42] Alham NK, Li M, Liu Y, Qi M. A MapReduce-based distributed SVM ensemble for scalable image classification and annotation. Computers & Mathematics with Applications 2013;66: 1920–1934.

[43] Wang D, Sun J Z, Li F C, Song T. Protein structure prediction based on parallel multi-class SVM. Application Research of Computers 2011; 28: 465–468.

[44] Cao JF, Li YF, Tian Y. Emotional modelling and classification of a large-scale collection of scene images in a cluster environment. PLOS ONE 2018; 13: 1–20. 10.1371/journal.pone.0191064 PMC5761962 29320579

