# Supplementary material for: Improved support vector machine classification algorithm based on adaptive feature weight updating in the Hadoop cluster environment
Source: PLoS One. 2019 Apr 10;14(4):e0215136. doi: 10.1371/journal.pone.0215136 (PMC6457544; doi:10.1371/journal.pone.0215136)
Supplement: S1 File — (DOC) [file pone.0215136.s001.doc]

**Clac_features_from_mat**

package fire;

import java.util.ArrayList;

import java.util.List;

import org.opencv.core.CvType;

import org.opencv.core.Mat;

import org.opencv.imgproc.Imgproc;

public class Clac_features_from_mat {

private Img_utils my_utils=null;

double y;

private List<String> features=null;

List<Double> this_feature=new ArrayList<Double>();

//String csv_save_feature;

public Clac_features_from_mat()

{

}

public void initilize()

{

my_utils=new Img_utils();

}

public Clac_features_from_mat(Mat mat,Double this_y)

{

this.initilize();

Img_preproces imgPro=new Img_preproces(mat);

//HUE feature

double[] hue=my_utils.get_Hue(imgPro.get_img(), imgPro.img_bin());

this_feature.add(new Double(hue[0]));

this_feature.add(new Double(hue[1]));

//Hu feature

Img_shape my_shape=new Img_shape(imgPro.img_gray());

double hu=my_shape.get_humoments();

//pca_sift feature

this_feature.addAll(my_shape.img_sift());

//Hu(8)

//My_Shape myshape = new My_Shape(mypreprocess.get_img_gray());

double[] Hu_8=my_shape.get_humoments_hu8();

for(int i=0;i<Hu_8.length;i++)

{

this_feature.add(Hu_8[i]);

}

//Extract PCA_Canny feature of the image

this_feature.addAll(my_shape.pca_canny_feature());

y=this_y;

}

public void save_feature(String csv_filename,List<String> features )

{

Img_csv my_csv=new Img_csv(csv_filename);

my_csv.write(features);

}

public void generate_feature_from_x_y(List<List<Double>> x,List<Double> y)

{

features=new ArrayList<String>();

for(int i=0;i<y.size();i++)

{

Integer this_y=new Integer(new Double(y.get(i)).intValue());

int category_int=this_y.intValue();

String this_line="";

this_line+=Integer.toString(category_int)+",";

for(Double this_feature:x.get(i))

{

this_line+=this_feature.toString()+",";

}

features.add(this_line);

}

}

public List<Double> get_x()

{

return this_feature;

}

public double get_y()

{

return y;

}

public List<String> get_features()

{

return features;

}

}

**Img_csv**

package fire;

import java.io.IOException;

import java.io.PrintWriter;

import java.net.URI;

import java.util.List;

import org.apache.commons.io.IOUtils;

import org.apache.hadoop.conf.Configuration;

import org.apache.hadoop.fs.FSDataOutputStream;

import org.apache.hadoop.fs.FileSystem;

import org.apache.hadoop.fs.Path;

public class Img_csv {

String csv_file;

public Img_csv(String csv_file)

{

this.csv_file=csv_file;

}

public void write(List<String> data)

{

//StringBuilder builder=new StringBuilder();

String total_line="";

for(String line:data)

{

total_line+=line+'\n';

//builder.append(line+'\n');

}

try{

Configuration conf = new Configuration();

FileSystem fs = FileSystem.get(URI.create("hdfs://master:9000"), conf);

Path file = new Path(csv_file);

FSDataOutputStream outStream = fs.create(file); // Creat a new file in a given path

//outStream.writeUTF(total_line);

outStream.write(total_line.getBytes());

outStream.close();

}catch(IOException e){

e.printStackTrace();

}

}

}

**Img_predict_data**

package fire;

import java.io.IOException;

import java.net.URI;

import java.util.ArrayList;

import java.util.List;

import org.apache.hadoop.conf.Configuration;

import org.apache.hadoop.fs.FSDataInputStream;

import org.apache.hadoop.fs.FileStatus;

import org.apache.hadoop.fs.FileSystem;

import org.apache.hadoop.fs.Path;

import org.opencv.core.Mat;

import bishe.Image.RawImage;

public class Img_predict_data {

private Img_utils my_utils=null;

private List<List<Double>> x=new ArrayList<List<Double>>();

private List<Double> y=new ArrayList<Double>();

List<Double> this_feature=null;

public void read_all_Mat(String dirpath) throws IOException

{

Configuration conf = new Configuration();//Get environmental variables

FileSystem fs = FileSystem.get(URI.create("hdfs://master:9000"), conf); // Create File System Instances

Path dir=new Path(dirpath); // Set a directory path

FileStatus[] fileList= fs.listStatus(dir);

for(FileStatus line:fileList)

{

FSDataInputStream in=fs.open(line.getPath());

RawImage img=new RawImage(in);

in.close();

Mat predict_data=img.toMat();

String[] split_line=line.getPath().toString().split("-|\\.");

String y_cat=split_line[1];

double y_category=Double.valueOf(y_cat);

Clac_features_from_mat(predict_data,y_category);

}

}

public void initilize()

{

my_utils=new Img_utils();

this_feature=new ArrayList<Double>();

}

public void Clac_features_from_mat(Mat mat ,double this_y)

{

this.initilize();

Img_preproces imgPro=new Img_preproces(mat);

double[] hue=my_utils.get_Hue(imgPro.get_img(), imgPro.img_bin());

this_feature.add(new Double(hue[0]));

this_feature.add(new Double(hue[1]));

Img_shape my_shape=new Img_shape(imgPro.img_gray());

//double hu=my_shape.get_humoments();

//this_feature.add((hu));

//PCA_SIFT feature

this_feature.addAll(my_shape.img_sift());

//Hu(8)

//My_Shape myshape = new My_Shape(mypreprocess.get_img_gray());

double[] Hu_8=my_shape.get_humoments_hu8();

for(int i=0;i<Hu_8.length;i++)

{

this_feature.add(Hu_8[i]);

}

//Extract PCA_SIFT feature of the image

this_feature.addAll(my_shape.pca_canny_feature());

x.add(this_feature);

y.add(this_y);

}

public List<List<Double>> get_predict_data()

{

return x;

}

public List<Double> get_category()

{

return y;

}

public static void main(String[] args) {

// TODO Auto-generated method stub

}

}

**Img_preproces**

package fire;

import org.opencv.core.Core;

import org.opencv.core.Mat;

import org.opencv.core.Size;

import org.opencv.imgproc.Imgproc;

public class Img_preproces {

private Mat img=null;

private Mat img_gray=null;

private Mat img_bin=null;

static

{

System.loadLibrary(Core.NATIVE_LIBRARY_NAME);

}

public Img_preproces(Mat mat)

{

this(mat,150.0,150.0);

}

public Img_preproces(Mat mat, double resize_width, double resize_height) {

// TODO Auto-generated constructor stub

this.img=new Mat();

Size s=new Size(resize_width,resize_height);

Imgproc.resize(mat, this.img, s, 0, 0, Imgproc.INTER_CUBIC);

this.filter();

this.rgb2gray();

this.gray2binary();

this.morphology(3.0);

this.path_img_bin_edge();

}

private void filter()

{

Mat mat =new Mat();

Imgproc.medianBlur(img, mat, 1);

Imgproc.bilateralFilter(mat, this.img, -1, 20.0, 20.0);

}

private void rgb2gray()

{

img_gray=new Mat();

Imgproc.cvtColor(this.img, img_gray, Imgproc.COLOR_BGR2GRAY);

}

private void gray2binary()

{

img_bin=new Mat();

Imgproc.threshold(this.img_gray, this.img_bin, 127.0, 255.0, Imgproc.THRESH_BINARY_INV+Imgproc.THRESH_OTSU);

}

private void morphology(double radius)

{

Size s=new Size(radius,radius);

Mat kernel =Imgproc.getStructuringElement(Imgproc.MORPH_ELLIPSE, s);

Mat mat=new Mat();

Imgproc.morphologyEx(this.img_bin, mat, Imgproc.MORPH_CLOSE, kernel);

img_bin=mat;

}

private void path_img_bin_edge()

{

int width = img_bin.cols();

int height = img_bin.rows();

for(int i=0;i<width;i++)

{

img_bin.put(0, i, (double)0.0);

img_bin.put(height-1, i, (double)0.0);

}

for (int i = 0; i < height; i++) {

img_bin.put(i, 0, (double) 0.0);

img_bin.put(i, width - 1, (double) 0.0);

}

}

public Mat get_img()

{

return img;

}

public Mat img_gray()

{

return img_gray;

}

public Mat img_bin()

{

return img_bin;

}

}

**Img_random**

package fire;

import java.util.ArrayList;

import java.util.List;

import java.util.Random;

public class Img_random {

public int[] randomArray(int min,int max,int n){

int len = max-min+1;

if(max < min || n > len){

return null;

}

// Initialize the selected array with a given range

int[] source = new int[len];

for (int i = min; i < min+len; i++){

source[i-min] = i;

}

int[] result = new int[n];

Random rd = new Random();

int index = 0;

for (int i = 0; i < result.length; i++) {

index = Math.abs(rd.nextInt() % len--);

result[i] = source[index];

source[index] = source[len];

}

return result;

}

}

**Img_rgroup_data**

package fire;

import java.util.ArrayList;

import java.util.List;

public class Img_rgroup_data {

List<List<List<Float>>> Features1_2=new ArrayList<List<List<Float>>>();

List<List<Double>> category1_2=new ArrayList<List<Double>>();

public void generate_rgroup_data(List<List<Float>> Features1,List<List<Float>> Features2, List<Double> category1, List<Double> category2,int num_group,int min, int max,int m)

{

for(int i=0;i<num_group;i++)

{

Img_svm_data kk=new Img_svm_data();

kk.get_Random_train_Features(Features1,category1,min,max,m);

kk.get_Random_train_Features(Features2,category2,min,max,m);

Features1_2.add(kk.get_svm_train_features());

category1_2.add(kk.get_svm_category());

}

}

public List<List<List<Float>>> get_train_features()

{

return Features1_2;

}

public List<List<Double>> get_train_category()

{

return category1_2;

}

public static void main(String[] args) {

// TODO Auto-generated method stub

}

}

**Img_shape**

package fire;

import java.util.ArrayList;

import java.util.List;

import org.opencv.core.Core;

import org.opencv.core.Mat;

import org.opencv.core.MatOfKeyPoint;

import org.opencv.core.MatOfPoint;

import org.opencv.features2d.DescriptorExtractor;

import org.opencv.features2d.FeatureDetector;

import org.opencv.imgproc.Imgproc;

import org.opencv.imgproc.Moments;

public class Img_shape {

Mat img=null;

double canny_thresh;

List<MatOfPoint> contours=null;

int area_largest_cnt_index=0;

Moments moment_largest=null;

List<Double> pca_canny=new ArrayList<Double>();

List<Double> pca_sift=new ArrayList<Double>();

static

{

System.loadLibrary(Core.NATIVE_LIBRARY_NAME);

}

public Img_shape(Mat img_gray)

{

this(img_gray,20.0);

}

public Img_shape(Mat img_gray, double canny_thresh) {

// TODO Auto-generated constructor stub

this.img=img_gray;

this.canny_thresh=canny_thresh;

this.find_largest_contours(20);

}

private void find_largest_contours(double thresh)

{

Mat edges=new Mat();

contours=new ArrayList<>();

Imgproc.Canny(this.img, edges, thresh, thresh*2);

Imgproc.findContours(edges, this.contours, new Mat(), Imgproc.RETR_TREE, Imgproc.CHAIN_APPROX_SIMPLE);

Mat mean = new Mat();

Mat vectors = new Mat();

Mat result=new Mat();

Mat Mat_nor=new Mat();

Core.normalize(edges, Mat_nor,-1.0, 1.0, Core.NORM_MINMAX);

Core.PCACompute(Mat_nor, mean, vectors,32);

Core.PCAProject(Mat_nor, mean, vectors, result);

Mat mean_2 = new Mat();

Mat vectors_2 = new Mat();

Mat result_2=new Mat();

Core.PCACompute(result.t(), mean_2, vectors_2,32);

Core.PCAProject(result.t(), mean_2, mean_2, result_2);

for(int i=0;i<result_2.rows();i++)

{

pca_canny.add(result_2.get(i, 0)[0]);

}

double area_largest=0.0;

double moment_area=0.0;

for(int i=0;i<contours.size();i++)

{

Moments moments=Imgproc.moments(contours.get(i));

if(moments.get_m00()!=0.0)

{

moment_area=moments.get_m00();

if(area_largest<moment_area)

{

area_largest=moment_area;

area_largest_cnt_index=i;

moment_largest=moments;

}

}

}

}

public double get_humoments()

{

Mat hu=new Mat();

Imgproc.HuMoments(this.moment_largest, hu);

double res=hu.get(0, 0)[0];

if(res<0.0)

{

return 0.0;

}

if(res>1.0)

{

return 1.0;

}

return res;

}

public double[] get_humoments_hu8() {

Moments p=this.moment_largest;

double

n20 = p.get_nu20(),

n02 = p.get_nu02(),

n30 = p.get_nu30(),

n12 = p.get_nu12(),

n21 = p.get_nu21(),

n03 = p.get_nu03(),

n11 = p.get_nu11();

double[] moments = new double[8];

//First moment

moments[0] = n20 + n02;

//Second moment

moments[1] = Math.pow((n20 - 02), 2) + Math.pow(2 * n11, 2);

//Third moment

moments[2] = Math.pow(n30 - (3 * (n12)), 2)

+ Math.pow((3 * n21 - n03), 2);

//Fourth moment

moments[3] = Math.pow((n30 + n12), 2) + Math.pow((n12 + n03), 2);

//Fifth moment

moments[4] = (n30 - 3 * n12) * (n30 + n12)

* (Math.pow((n30 + n12), 2) - 3 * Math.pow((n21 + n03), 2))

+ (3 * n21 - n03) * (n21 + n03)

* (3 * Math.pow((n30 + n12), 2) - Math.pow((n21 + n03), 2));

//Sixth moment

moments[5] = (n20 - n02)

* (Math.pow((n30 + n12), 2) - Math.pow((n21 + n03), 2))

+ 4 * n11 * (n30 + n12) * (n21 + n03);

//Seventh moment

moments[6] = (3 * n21 - n03) * (n30 + n12)

* (Math.pow((n30 + n12), 2) - 3 * Math.pow((n21 + n03), 2))

+ (n30 - 3 * n12) * (n21 + n03)

* (3 * Math.pow((n30 + n12), 2) - Math.pow((n21 + n03), 2));

//Eighth moment

moments[7] = n11 * (Math.pow((n30 + n12), 2) - Math.pow((n03 + n21), 2))

- (n20 - n02) * (n30 + n12) * (n03 + n21);

return moments;

}

public List<Double> img_sift()

{

Mat siftMat=new Mat();

FeatureDetector fd = FeatureDetector.create(FeatureDetector.SURF);

MatOfKeyPoint mkp =new MatOfKeyPoint();

fd.detect(this.img, mkp);

DescriptorExtractor de = DescriptorExtractor.create(DescriptorExtractor.SURF);

de.compute(this.img,mkp,siftMat );

Mat mean = new Mat();

Mat vectors = new Mat();

Mat result=new Mat();

Mat siftMat_r=new Mat();

Core.normalize(siftMat, siftMat_r,-1.0, 1.0, Core.NORM_MINMAX);

Core.PCACompute(siftMat_r, mean, vectors,64);

Core.PCAProject(siftMat_r, mean, vectors, result);

Mat mean_2 = new Mat();

Mat vectors_2 = new Mat();

Mat result_2=new Mat();

Core.PCACompute(result.t(), mean_2, vectors_2,64);

Core.PCAProject(result.t(), mean_2, mean_2, result_2);

for(int j=0;j<result_2.rows();j++)

{

pca_sift.add(result_2.get(j, 0)[0]);

}

int sift_mat_size=pca_sift.size();

while(sift_mat_size!=64)

{

pca_sift.add(0.0);

sift_mat_size=sift_mat_size+1;

}

return pca_sift;

}

public List<Double> pca_canny_feature()

{

return pca_canny;

}

}

**Img_svm**

package fire;

import java.util.ArrayList;

import java.util.List;

import org.apache.hadoop.io.Text;

import org.opencv.core.*;

import org.opencv.ml.CvSVM;

import org.opencv.ml.CvSVMParams;

public class Img_svm extends StatModel{

static

{

System.loadLibrary(Core.NATIVE_LIBRARY_NAME);

}

private CvSVM model_svm=null;

final private String trained_model_filename="home/ubuntu01/svm_opencv_train/cv2_svm_model.xml";

private CvSVMParams my_svm_params=new CvSVMParams();

public Img_svm(double svm_param_C,double svm_param_gamma)

{

System.out.println("C,gamma= "+svm_param_C+", "+svm_param_gamma);

this.model_svm=new CvSVM();

this.my_svm_params.set_svm_type(CvSVM.C_SVC);

this.my_svm_params.set_kernel_type(CvSVM.RBF);

this.my_svm_params.set_C(svm_param_C);

this.my_svm_params.set_gamma(svm_param_gamma);

}

public void train(List<List<Float>> train_feature,List<Double> train_category)

{

int item_features_qty=train_feature.get(0).size();

int item_qty=train_category.size();

Mat mat_train_x=new Mat(item_qty,item_features_qty,CvType.CV_32FC1);

Mat mat_train_y=new Mat(item_qty,1,CvType.CV_32FC1);

for(int i=0;i<item_qty;i++)

{

mat_train_y.put(i, 0, train_category.get(i).floatValue() );

for(int j=0;j<item_features_qty;j++)

{

mat_train_x.put(i, j, train_feature.get(i).get(j));

}

}

model_svm.train(mat_train_x, mat_train_y,new Mat(),new Mat(),my_svm_params);

}

public List<Double> predict(List<List<Double>> predict_feature)

{

int item_features_qty=predict_feature.get(0).size();

int item_qty=predict_feature.size();

Mat mat_predict_x=new Mat(item_qty,item_features_qty,CvType.CV_32FC1);

Mat mat_predice_y=new Mat(item_qty,1,CvType.CV_32FC1);

for(int i=0;i<item_qty;i++)

{

for(int j=0;j<item_features_qty;j++)

{

mat_predict_x.put(i, j,predict_feature.get(i).get(j).floatValue() );

}

}

model_svm.predict_all(mat_predict_x, mat_predice_y);

List<Double> result =new ArrayList<Double>();

for(int i=0;i<mat_predice_y.rows();i++)

{

result.add(mat_predice_y.get(i,0)[0]);

}

return result;

}

public void save()

{

this.save(trained_model_filename);

}

public void save(String filename)

{

model_svm.save(filename);

}

public static void main(String[] args) {

// TODO Auto-generated method stub

}

}

**Img_svm_data**

package fire;

import java.util.ArrayList;

import java.util.List;

public class Img_svm_data {

private List<List<Float>> svm_train_features=new ArrayList<List<Float>>();

private List<Float> svm_test_features=new ArrayList<Float>();

private List<Double> svm_train_category=new ArrayList<Double>();

public void get_Random_train_Features(List<List<Float>> features,List<Double> train_category,int min, int max,int m)

{

Img_random img_rad=new Img_random();

int [] Img_rad=img_rad.randomArray(min, max, m);

for(int i=0;i<Img_rad.length;i++)

{

svm_train_features.add(features.get(Img_rad[i]));

svm_train_category.add(train_category.get(Img_rad[i]));

}

}

public void get_Random_test_Features(List<Float> features1,List<Float> features2)

{

Img_random img_rad=new Img_random();

int [] Img_rad=img_rad.randomArray(1, 10, 4);

for(int i=0;i<Img_rad.length;i++)

{

svm_test_features.add(features1.get(Img_rad[i]));

svm_test_features.add(features2.get(Img_rad[i]));

}

}

public List<List<Float> >get_svm_train_features()

{

return svm_train_features;

}

public List<Float> get_svm_test_features()

{

return svm_test_features;

}

public List<Double> get_svm_category()

{

return svm_train_category;

}

public static void main(String[] args) {

// TODO Auto-generated method stub

Img_random img_rad=new Img_random();

int [] Img_rad=img_rad.randomArray(0, 9,6);

for (int i : Img_rad) {

System.out.println(i);

}

}

}

**Img_utils**

package fire;

import org.opencv.core.Mat;

import org.opencv.imgproc.Imgproc;

public class Img_utils {

public Img_utils()

{

}

public double[] get_Hue(Mat input_img, Mat input_img_bin)

{

int total_vaild_pixels=0;

int sum_H=0;

int height=input_img_bin.rows();

int width=input_img_bin.cols();

Mat img_hsv=new Mat();

Imgproc.cvtColor(input_img, img_hsv, Imgproc.COLOR_BGR2HSV);

for(int y=0; y< height; y++)

{

for(int x=0; x< width; x++)

{

if(input_img_bin.get(y, x)[0]>0)

{

total_vaild_pixels+=1;

double[] this_px=img_hsv.get(y, x);

sum_H +=new Double(this_px[0]).intValue();

}

}

}

double sum_H_avg =(new Double(sum_H).doubleValue())/total_vaild_pixels;

double sum_H_avg_periodically=Math.PI*sum_H_avg/90;

return (new double[] {(1+Math.cos(sum_H_avg_periodically))/2,(1+Math.sin(sum_H_avg_periodically))/2});

}

}

package fire;

import java.util.ArrayList;

import java.util.Arrays;

import java.util.HashMap;

import java.util.List;

import java.util.Map;

import java.util.TreeSet;

import org.opencv.core.Core;

import org.opencv.core.Mat;

import org.opencv.imgproc.Imgproc;

**My_RILBP**

public class My_RILBP {

static {

System.loadLibrary(Core.NATIVE_LIBRARY_NAME);

}

static double lbp_radius;

static int lbp_neighbors;

static Map<Integer, Integer> dict_sum_to_rilbp = null;

static Map<Integer, Integer> dict_rilbp_to_histogram_x = null;

static int histogram_x_width = 0;

int[] histogram_result = null;

Mat img = null;

public My_RILBP() {

this(1.0, 8);

}

public My_RILBP(double radius, int neighbors) {

if (My_RILBP.dict_sum_to_rilbp == null) {

// System.out.println("generating...");

My_RILBP.lbp_radius = radius;

My_RILBP.lbp_neighbors = neighbors;

My_RILBP.gen_dict_sum_to_rilbp();

My_RILBP.gen_dict_rilbp_to_histogram_x();

}

}

static private void gen_dict_sum_to_rilbp() {

int max_val = 1 << lbp_neighbors;

dict_sum_to_rilbp = new HashMap<Integer, Integer>();

for (int i = 0; i < max_val; i++) {

List<Integer> bits = new ArrayList<Integer>();

for (int bit = 0; bit < lbp_neighbors; bit++) {

bits.add(new Integer(i & (1 << bit)) >> bit);

}

int sum_ = get_rilbp_from_bin(bits);

dict_sum_to_rilbp.put(i, sum_);

}

}

static private int get_sum_from_bin(List<Integer> input_list) {

int res = 0;

int len = input_list.size();

for (int i = 0; i < len; i++) {

res += (input_list.get(i).intValue() << (i));

}

return res;

}

static private int get_rilbp_from_bin(List<Integer> input_list) {

int len = input_list.size();

int min_lbp = get_sum_from_bin(input_list);

int this_sum;

for (int i = 1; i < len; i++) {

Integer first_element = input_list.remove(0);

input_list.add(first_element);

this_sum = get_sum_from_bin(input_list);

if (this_sum < min_lbp)

min_lbp = this_sum;

}

return min_lbp;

}

static private void gen_dict_rilbp_to_histogram_x() {

TreeSet<Integer> set_rilbp = new TreeSet<Integer>();

dict_rilbp_to_histogram_x = new HashMap<Integer, Integer>();

for (Map.Entry<Integer, Integer> entry : dict_sum_to_rilbp.entrySet()) {

set_rilbp.add(entry.getValue());

}

Integer index = new Integer(0);

for (Integer i : set_rilbp) {

dict_rilbp_to_histogram_x.put(i, index);

index += 1;

}

histogram_x_width = index;

}

private double get_pixel_else_0(double idx, double idy) {

int x = new Double(idx).intValue();

int y = new Double(idy).intValue();

if (x < img.cols() && y < img.rows())

return img.get(y, x)[0];

else

return 0.0;

}

private double bilinear_interpolation(double x, double y) {

double x1 = new Double(x).intValue();

double y1 = new Double(y).intValue();

double x2 = Math.ceil(x);

double y2 = Math.ceil(y);

double r1 = (x2 - x) / (x2 - x1) * get_pixel_else_0(x1, y1) + (x - x1) / (x2 - x1) * get_pixel_else_0(x2, y1);

double r2 = (x2 - x) / (x2 - x1) * get_pixel_else_0(x1, y2) + (x - x1) / (x2 - x1) * get_pixel_else_0(x2, y2);

return (y2 - y) / (y2 - y1) * r1 + (y - y1) / (y2 - y1) * r2;

}

private boolean is_int_equal_double(double num) {

if (Math.abs(num - (new Double(num).intValue())) <= 1E-17)

return true;

else

return false;

}

static public Object get_d() {

return dict_rilbp_to_histogram_x;

}

public List<Double> get_lbp_histogram(Mat input_img) {

if (input_img.channels() != 1) {

img = new Mat();

Imgproc.cvtColor(input_img, img, Imgproc.COLOR_RGB2GRAY);

} else {

img = input_img;

}

histogram_result = new int[My_RILBP.histogram_x_width];

Arrays.fill(histogram_result, 0);

double r1, r2, c1, c2, w1, w2, res;

int rilbp_sum, rilbp_min, histogram_x;

for (int x = 0; x < img.cols(); x++) {

for (int y = 0; y < img.rows(); y++) {

double center = get_pixel_else_0(x, y);

List<Double> pixels = new ArrayList<Double>();

for (int point = 1; point <= My_RILBP.lbp_neighbors; point++) {

double r = x + My_RILBP.lbp_radius * Math.cos(2 * Math.PI * point / My_RILBP.lbp_neighbors);

double c = y - My_RILBP.lbp_radius * Math.sin(2 * Math.PI * point / My_RILBP.lbp_neighbors);

if (r < 0.0 || c < 0.0) {

pixels.add(0.0);

continue;

}

if (is_int_equal_double(r)) {

if (is_int_equal_double(c) == false) {

c1 = new Double(c).intValue();

c2 = Math.ceil(c);

w1 = (c2 - c) / (c2 - c1);

w2 = (c - c1) / (c2 - c1);

res = w1 * get_pixel_else_0(r, c) + w2 * get_pixel_else_0(r, Math.ceil(c));

res = res / (w1 + w2);

pixels.add(new Double(res));

} else {

pixels.add(get_pixel_else_0(r, c));

}

} else if (is_int_equal_double(c)) {

r1 = new Double(r).intValue();

r2 = Math.ceil(r);

w1 = (r2 - r) / (r2 - r1);

w2 = (r - r1) / (r2 - r1);

res = w1 * get_pixel_else_0(r, c) + w2 * get_pixel_else_0(Math.ceil(r), c);

res = res / (w1 + w2);

pixels.add(new Double(res));

} else {

pixels.add(bilinear_interpolation(r, c));

}

}

List<Integer> value_bits = thresholded(center, pixels);

rilbp_sum = get_sum_from_bin(value_bits);

rilbp_min = dict_sum_to_rilbp.get(rilbp_sum);

histogram_x = dict_rilbp_to_histogram_x.get(rilbp_min);

histogram_result[histogram_x] += 1;

}

}

double histogram_y_sum = 0;

for (int i : histogram_result) {

histogram_y_sum += i;

}

List<Double> histogram_y_normalized = new ArrayList<Double>();

for (int i : histogram_result)

histogram_y_normalized.add(new Double(i / histogram_y_sum));

return histogram_y_normalized;

}

private List<Integer> thresholded(double center, List<Double> pixels) {

List<Integer> out = new ArrayList<Integer>();

for (Double i : pixels) {

if (i.doubleValue() >= center)

out.add(1);

else

out.add(0);

}

return out;

}

}

**Image**

package bishe;

import java.io.DataInput;

import java.io.DataOutput;

import java.io.File;

import java.io.FileInputStream;

import java.io.IOException;

import java.io.InputStream;

import org.apache.hadoop.io.Writable;

import org.opencv.core.Core;

import org.opencv.core.CvType;

import org.opencv.core.Mat;

import org.opencv.core.MatOfByte;

import org.opencv.highgui.Highgui;

public class Image {

public static class RawImage implements Writable {

static {

System.loadLibrary(Core.NATIVE_LIBRARY_NAME);

}

private byte[] rawdata;

public RawImage() {

// TODO Auto-generated constructor stub

rawdata = null;

}

public RawImage(InputStream in) throws IOException {

int off = 0;

int readBytes = 0;

int totalBytes = in.available();

rawdata = new byte[totalBytes];

while( (readBytes = in.read(rawdata, off, totalBytes - off) ) > 0) {

off += readBytes;

}

if(off != totalBytes) {

String err = String.format("read %d bytes but total bytes is %d", off, totalBytes);

throw new IOException(err);

}

}

public RawImage(String filePath) throws IOException {

FileInputStream in = new FileInputStream(new File(filePath));

int fileSize = in.available();

rawdata = new byte[fileSize];

in.read(rawdata);

in.close();

}

public void set(byte[] data) {

rawdata = data;

}

@Override

public void readFields(DataInput input) throws IOException {

// TODO Auto-generated method stub

int fileSize = input.readInt();

rawdata = new byte[fileSize];

input.readFully(rawdata);

}

@Override

public void write(DataOutput output) throws IOException {

// TODO Auto-generated method stub

output.writeInt(rawdata.length);

output.write(rawdata);

}

public int getSize() {

if (rawdata == null) {

return 0;

} else {

return rawdata.length;

}

}

public byte[] getRawData() {

return rawdata;

}

public Mat toMat(int flags) {

Mat img = new Mat();

if (rawdata == null) {

return img;

} else {

MatOfByte buff = new MatOfByte(rawdata);

img = Highgui.imdecode(buff, flags);

return img;

}

}

public Mat toMat() {

return toMat(Highgui.CV_LOAD_IMAGE_COLOR);

}

public static RawImage toImage(Mat mat, String ext) {

RawImage img = new RawImage();

if(mat == null) {

return img;

} else {

MatOfByte buff = new MatOfByte();

Highgui.imencode(ext, mat, buff);

img.set(buff.toArray());

return img;

}

}

public static RawImage toImage(Mat mat) {

return toImage(mat, ".jpg");

}

}

public static class MatImage extends Mat implements Writable {

static {

System.loadLibrary(Core.NATIVE_LIBRARY_NAME);

}

public MatImage() {

// TODO Auto-generated constructor stub

super();

}

public MatImage(Mat mat) {

super(mat.nativeObj);

}

public MatImage(int rows, int cols, int type) {

super(rows, cols, type);

}

@Override

public void readFields(DataInput input) throws IOException {

// TODO Auto-generated method stub

int rows = input.readInt();

int cols = input.readInt();

int type = input.readInt();

int channels = CvType.channels(type);

int byteCnt = CvType.ELEM_SIZE(type) / channels;

create(rows, cols, type);

byte[] byteBuff = new byte[channels];

short[] shortBuff = new short[channels];

float[] floatBuff = new float[channels];

double[] doubleBuff = new double[channels];

for (int row = 0; row < rows; row++) {

for (int col = 0; col < cols; col++) {

if (byteCnt == 1) {

for (int i = 0; i < channels; i++) {

byteBuff[i] = input.readByte();

}

put(row, col, byteBuff);

} else if (byteCnt == 2) {

for (int i = 0; i < channels; i++) {

shortBuff[i] = input.readShort();

}

put(row, col, shortBuff);

} else if (byteCnt == 4) {

for (int i = 0; i < channels; i++) {

floatBuff[i] = input.readFloat();

}

put(row, col, floatBuff);

} else if (byteCnt == 8) {

for (int i = 0; i < channels; i++) {

doubleBuff[i] = input.readDouble();

}

put(row, col, doubleBuff);

} else {

throw new java.lang.UnsupportedOperationException(

"Unsupported CvType value: " + type);

}

}

}

}

@Override

public void write(DataOutput output) throws IOException {

// TODO Auto-generated method stub

output.writeInt(rows());

output.writeInt(cols());

output.writeInt(type());

int type = type();

int channels = channels();

int byteCnt = CvType.ELEM_SIZE(type) / channels;// (byte:1;short:2;float:4;double:8)

int rows = rows();

int cols = cols();

byte[] byteBuff = new byte[channels];

short[] shortBuff = new short[channels];

float[] floatBuff = new float[channels];

double[] doubleBuff = new double[channels];

for (int row = 0; row < rows; row++) {

for (int col = 0; col < cols; col++) {

if (byteCnt == 1) {

get(row, col, byteBuff);

for (int i = 0; i < channels; i++) {

output.writeByte(byteBuff[i]);

}

} else if (byteCnt == 2) {

get(row, col, shortBuff);

for (int i = 0; i < channels; i++) {

output.writeShort(shortBuff[i]);

}

} else if (byteCnt == 4) {

get(row, col, floatBuff);

for (int i = 0; i < channels; i++) {

output.writeFloat(floatBuff[i]);

}

} else if (byteCnt == 8) {

get(row, col, doubleBuff);

for (int i = 0; i < channels; i++) {

output.writeDouble(doubleBuff[i]);

}

} else {

throw new java.lang.UnsupportedOperationException(

"Unsupported CvType value: " + type);

}

}

}

}

}

public static void main(String[] args) throws IOException {

// TODO Auto-generated method stub

}

}

**Image_Readuce_Svm**

package bishe;

import java.io.IOException;

import java.util.ArrayList;

import java.util.HashMap;

import java.util.Iterator;

import java.util.List;

import java.util.Map;

import org.apache.commons.collections.IteratorUtils;

import org.apache.hadoop.io.DoubleWritable;

import org.apache.hadoop.io.IntWritable;

import org.apache.hadoop.io.Text;

import org.apache.hadoop.mapreduce.ReduceContext;

import org.apache.hadoop.mapreduce.Reducer;

import fire.Img_predict_data;

import fire.Img_rgroup_data;

import fire.Img_svm;

public class Image_Readuce_Svm extends Reducer <Text,DoubleWritable,Text,DoubleWritable>{

List<Double> accuracy=new ArrayList<Double>();

Double max=0.0;

protected void setup(Context context) throws IOException, InterruptedException {

System.out.println("1111111111111");

}

protected void reduce(Text key, Iterable<DoubleWritable> values, Context context

) throws IOException, InterruptedException {

System.out.println("key: "+key.toString());

for(DoubleWritable val:values)

{

if(val.get()>max)

{

max=val.get();

}else

{

break;

}

System.out.println("values: "+val.toString());

context.write(key, new DoubleWritable(max));

}

}

protected void cleanup(Context context) throws IOException, InterruptedException {

}

public static void main(String[] args) {

// TODO Auto-generated method stub

}

}

**ImageMapper**

package bishe;

import java.io.IOException;

import java.text.SimpleDateFormat;

import java.util.ArrayList;

import java.util.Date;

import java.util.List;

import org.apache.hadoop.fs.Path;

import org.apache.hadoop.io.DoubleWritable;

import org.apache.hadoop.io.IntWritable;

import org.apache.hadoop.io.NullWritable;

import org.apache.hadoop.io.SequenceFile;

import org.apache.hadoop.io.Text;

import org.apache.hadoop.mapreduce.Job;

import org.apache.hadoop.mapreduce.Mapper;

import org.apache.hadoop.mapreduce.Mapper.Context;

import org.apache.hadoop.mapreduce.Reducer;

import org.apache.hadoop.mapreduce.lib.input.FileInputFormat;

import org.apache.hadoop.mapreduce.lib.input.SequenceFileInputFormat;

import org.apache.hadoop.mapreduce.lib.input.TextInputFormat;

import org.apache.hadoop.mapreduce.lib.output.FileOutputFormat;

import org.apache.hadoop.mapreduce.lib.output.LazyOutputFormat;

import org.apache.hadoop.mapreduce.lib.output.MultipleOutputs;

import org.apache.hadoop.mapreduce.lib.output.TextOutputFormat;

import org.opencv.core.Core;

import org.opencv.core.CvType;

import org.opencv.core.Mat;

import org.opencv.imgproc.Imgproc;

import bishe.Image.RawImage;

import fire.Clac_features_from_mat;

import fire.Img_svm;

public class ImageMapper {

public static class RGB2GrayMapper extends Mapper<Object, Text, Text, DoubleWritable> {

private List<List<Double>> x=new ArrayList<List<Double>>();

List<List<Float>> feature_1=new ArrayList<List<Float>>();

List<List<Float>> feature_2=new ArrayList<List<Float>>();

List<List<Float>> feature_all=new ArrayList<List<Float>>();

List<List<Float>> feature_train=new ArrayList<List<Float>>();

List<List<Float>> feature_test=new ArrayList<List<Float>>();

List<Float> random_label_train=new ArrayList<Float>();

List<Float> random_label_test=new ArrayList<Float>();

cate_feature ca_f=null;

List<Float> commen_label=new ArrayList<Float>();

random_group rg=new random_group();

private List<Double> accuracy=new ArrayList<Double>();

protected void setup(Context context) throws IOException, InterruptedException

{

super.setup(context);

context.getInputSplit();

rg.get_random_g(6);

System.out.println("rg.get_group():"+rg.get_group().toString());

System.out.println("123123");

System.out.println("context.getInputSplit().toString(): "+context.getInputSplit().toString());

System.out.println("getJobID: "+context.getJobID());

}

@Override

protected void map(Object key, Text value, Context context) throws IOException, InterruptedException {

String[] feature_cate=value.toString().split("\\s");

String feature_x=value.toString().substring(4, value.toString().length());

String cate_y=feature_cate[0];

System.out.println("cate_y: "+cate_y);

if(Double.valueOf(cate_y)==1234567)

{

feature_all.addAll(feature_1);

feature_all.addAll(feature_2);

for(int i=0;i<6;i++)

{

random_feature rf=new random_feature();

rf.generate_random_feature(feature_all, commen_label, rg.get_group().get(i));

random_train_test rtt=new random_train_test();

rtt.random_train_test_feature(rf.get_feature_all(), rf.get_random_label());

feature_train= rtt.get_feature_train();

random_label_train=rtt.get_label_train();

Img_svm my_svm=new Img_svm(8.0, 0.0078125);

my_svm.train(feature_train, random_label_train);

Date date= new Date();

SimpleDateFormat sdf = new SimpleDateFormat("HH:mm:ss:SS");

String file_path = sdf.format(date);

String Map_out="cv2_svm_model_group_"+file_path+".xml"+rg.get_group().get(i).toString();

my_svm.save("/home/ubuntu01/svm_opencv_train/"+"cv2_svm_model_group_"+file_path+".xml");

List<Double> predict_y=my_svm.predict(rtt.get_feature_ttest());

int predict_error_counter=0;

for(int j=0;j<rtt.get_feature_ttest().size();j++)

{

double test_y_=rtt.get_label_test().get(j);

double predict_val=predict_y.get(j);

if(Math.abs(predict_val-test_y_)>0.01)

{

System.out.format("[WRONG] predict=%f, test_y=%f\n", predict_val,test_y_);

predict_error_counter+=1;

}

}

accuracy.add((double)(rtt.get_feature_ttest().size() - predict_error_counter) / rtt.get_feature_ttest().size());

System.out.format("train_num=%d, test_num=%d, predict_correct_num=%d\n",rtt.get_label_train().size(), rtt.get_label_test().size(), rtt.get_label_test().size() - predict_error_counter);

System.out.format("predict accuracy=%.1f%%\n", (100 * (double)(rtt.get_label_test().size() - predict_error_counter) / rtt.get_label_test().size()));

System.out.println("rg.get_group(): "+rg.get_group().toString());

System.out.println("predict_y: "+predict_y.toString());

System.out.println("feature_all: "+feature_all.size());

System.out.println("random_label: "+commen_label.size());

System.out.println("feature_train: "+feature_train.size());

System.out.println("random_label_train: "+random_label_train.toString());

System.out.println("random_label_test: "+rtt.get_label_test().toString());

Text Map_123=new Text();

Map_123.set(Map_out);

System.out.println("accuracy.get(i):"+accuracy.get(i));

System.out.println("Map_out: "+Map_out);

context.write(Map_123, new DoubleWritable(accuracy.get(i)));

}

}else

{

ca_f=new cate_feature();

ca_f.gengerate_cate_feature(cate_y, feature_x);

if(Double.valueOf(cate_y)==1)

{

feature_1.add(ca_f.get_x1());

commen_label.add((float) 1.0);

}else

{

feature_2.add(ca_f.get_x2());

commen_label.add((float) 2.0);

}

System.out.println("feature_1: "+feature_1.size());

System.out.println("feature_2: "+feature_2.size());

}

}

protected void cleanup(Context context) throws IOException, InterruptedException

{

}

}

public static void main(String[] args) throws IOException, ClassNotFoundException, InterruptedException {

// TODO Auto-generated method stub

Job job = Job.getInstance();

job.setJobName("ImageOutputTest");

job.setOutputKeyClass(Text.class);

job.setOutputValueClass(DoubleWritable.class);

job.setMapperClass(RGB2GrayMapper.class);

job.setReducerClass(Image_Readuce_Svm.class);

job.setCombinerClass(Reducer.class);

FileInputFormat.addInputPath(job, new Path("hdfs://master:9000/output4"));

FileOutputFormat.setOutputPath(job, new Path("hdfs://master:9000/output6"));

//FileInputFormat.setMaxInputSplitSize(job, 10712);

//FileInputFormat.setMinInputSplitSize(job, 1);

System.out.println("456456");

job.waitForCompletion(true);

}

}

**random_feature**

package bishe;

import java.util.ArrayList;

import java.util.List;

public class random_feature {

List<List<Float>> feature_all=new ArrayList<List<Float>>();

List<Float> feature_all_one=null;

List<Float> radom_label=new ArrayList<Float>();

public void generate_random_feature(List<List<Float>> feature,List<Float> label,List<Integer> randomq)

{

for(int k=0;k<feature.size();k++)

{

feature_all_one = new ArrayList<Float>();

feature_all_one.add(randomq.get(0)*feature.get(k).get(0));

feature_all_one.add(randomq.get(0)*feature.get(k).get(1));

for(int i=2;i<=33;i++)

{

feature_all_one.add(randomq.get(1)*feature.get(k).get(i));

}

for(int i=34;i<=97;i++)

{

feature_all_one.add(randomq.get(2)*feature.get(k).get(i));

}

feature_all.add(feature_all_one);

radom_label.add(label.get(k));

}

}

public List<List<Float>> get_feature_all()

{

return feature_all;

}

public List<Float> get_random_label()

{

return radom_label;

}

}

**random_group**

package bishe;

import java.util.ArrayList;

import java.util.List;

import java.util.Random;

public class random_group {

List<List<Integer>> ss=new ArrayList<List<Integer>>();

public void get_random_g(int n)

{

List<Integer> tt=null;

for(int i=0;i<n;i++)

{

tt=new ArrayList<Integer>();

int max=8;

int min=1;

Random random = new Random();

int A = random.nextInt(max)%(max-min+1) + min;

tt.add(A);

int B =random.nextInt(10-A)%((10-A)-min+1)+min;

while(A+B==10||A+B>=10)

{

int B_1 =random.nextInt(10-A)%((10-A)-min+1)+min;

B=B_1;

}

tt.add(B);

int C=10-(A+B);

tt.add(C);

ss.add(tt);

}

}

public List<List<Integer>> get_group()

{

return ss;

}

}
