# Supplementary material for: Improved support vector machine classification algorithm based on adaptive feature weight updating in the Hadoop cluster environment
Source: PLoS One. 2019 Apr 10;14(4):e0215136. doi: 10.1371/journal.pone.0215136 (PMC6457544; doi:10.1371/journal.pone.0215136)
Supplement: S2 File — (DOC) [file pone.0215136.s002.doc]

**S2 file.** Training time and classification accuracy comparisons of different algorithms on the ImageNet dataset.

1. Training time

Training time comparison results of the method proposed in this paper and those of CNN, AlexNet, VGGNet, GoogleNet, ResNet and DenseNet are shown in Supplementary Figure 1.


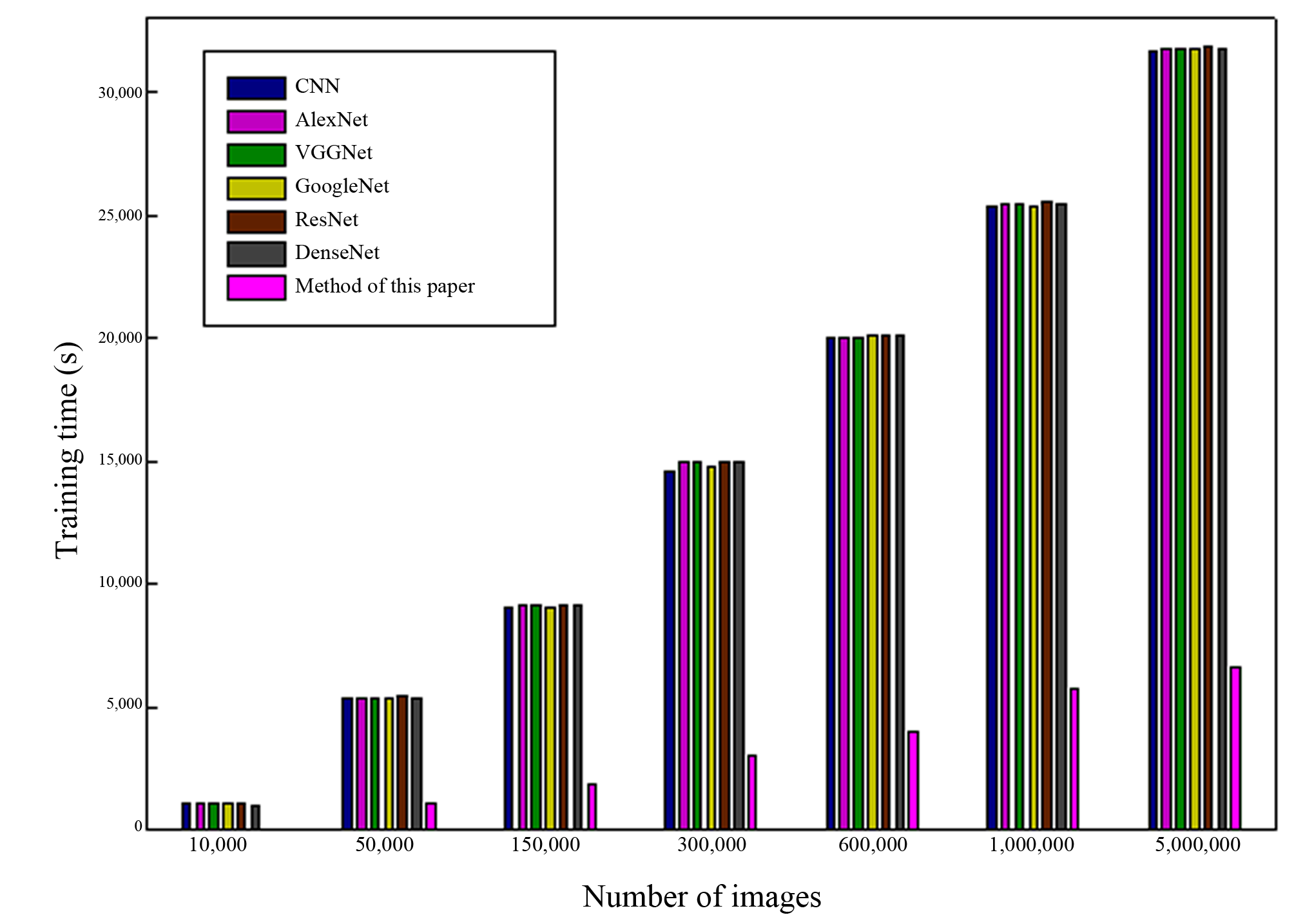


**Supplementary Figure 1**. Training time comparison for different algorithms on the ImageNet image library.

The results in Supplementary Figure 1 clearly show that although the training time of all algorithms increases as the number of images increases, the training time of the six deep learning algorithms is obviously much greater than that of the method proposed in this paper. This result further illustrates that regardless of the image type, deep learning algorithms will require long training times in a CPU environment due to the complexity of the training process itself. The method proposed in this paper is based on parallel extraction of the specified features in a distributed environment and uses the MapReduce parallel programming model for parallel classification. Consequently, the training time for the method proposed in this paper is much shorter than that for the other deep learning algorithms, which also illustrates the shortcomings of deep learning algorithms in a CPU environment.

2. Classification accuracy

Supplementary Table 1 lists the results of the classification accuracy comparison with different algorithms.

**Supplementary Table 1**. Classification accuracy comparison of different algorithms on the ImageNet image library

| Number of images | Image categories | Classification accuracy (%) | | | | | | |
| --- | --- | --- | --- | --- | --- | --- | --- | --- |
|  |  | CNN | AlexNet | VGGNet | GoogleNet | ResNet | DenseNet | Method of this paper |
| 10,000 | 20 | 96.83 | 96.82 | 96.84 | 96.85 | 96.84 | 96.86 | 96.85 |
|  | 50 | 96.83 | 96.81 | 96.85 | 96.85 | 96.84 | 96.86 | 96.85 |
|  | 100 | 96.82 | 96.81 | 96.84 | 96.84 | 96.83 | 96.85 | 96.84 |
| 50,000 | 100 | 95.27 | 95.27 | 95.26 | 95.29 | 95.29 | 95.30 | 95.29 |
|  | 500 | 95.28 | 95.27 | 95.25 | 95.29 | 95.29 | 95.31 | 95.29 |
|  | 1,000 | 95.27 | 95.27 | 95.25 | 95.28 | 95.29 | 95.29 | 95.29 |
| 150,000 | 300 | 95.14 | 95.13 | 95.13 | 95.15 | 95.15 | 95.17 | 95.14 |
|  | 500 | 95.15 | 95.13 | 95.12 | 95.16 | 95.15 | 95.16 | 95.14 |
|  | 1,500 | 95.14 | 95.13 | 95.13 | 95.15 | 95.14 | 95.16 | 95.13 |
| 300,000 | 500 | 95.02 | 95.04 | 95.03 | 95.06 | 95.05 | 95.06 | 95.02 |
|  | 1,000 | 95.02 | 95.03 | 95.03 | 95.05 | 95.04 | 95.06 | 95.02 |
|  | 2,000 | 95.02 | 95.03 | 95.03 | 95.05 | 95.05 | 95.06 | 95.02 |
| 600,000 | 1,000 | 94.68 | 94.68 | 94.71 | 94.72 | 94.69 | 94.74 | 94.69 |
|  | 2,000 | 94.68 | 94.67 | 94.71 | 94.72 | 94.70 | 94.74 | 94.67 |
|  | 5,000 | 94.66 | 94.66 | 94.70 | 94.70 | 94.69 | 94.74 | 94.69 |
| 1,000,000 | 2,000 | 94.17 | 94.18 | 94.20 | 94.20 | 94.18 | 94.22 | 94.17 |
|  | 5,000 | 94.17 | 94.17 | 94.19 | 94.20 | 94.19 | 94.20 | 94.17 |
| 5,000,000 | 5,000 | 93.55 | 93.54 | 93.58 | 93.58 | 93.57 | 93.59 | 93.56 |
|  | 8,350 | 93.56 | 93.54 | 93.57 | 93.56 | 93.55 | 93.59 | 93.55 |

From the experimental data in Supplementary Table 1, it can be seen that as the number of images from the ImageNet dataset increases, the classification accuracy of various algorithms decreases, but the downward trend is subtle. This result fully demonstrates the advantages of distributed clustered systems and deep learning frameworks, which are capable of adequately training on large numbers of images to achieve optimal classification performance. In addition, under the same number of datasets, the image classification accuracy is not very high given the number of image categories; moreover, the classification accuracy remains basically the same regardless of the number of categories, although it is sometimes slightly reduced or increased, which is related to the complexity of the selected image itself. These results fully demonstrate that the seven algorithms have sufficient learning ability to learn the main content of the images during the training process. Although the approach in this paper involves extracting features manually, its classification accuracy is almost the same as that of other deep learning algorithms because it selects the most commonly used features that best express the image content. This result further demonstrates that the proposed method is suitable for experimental sites with limited hardware support and that it achieves better classification performance while reducing hardware costs and training time. Other scientific researchers will be able to capitalize on both this method and its underlying concepts.
